# Experience of social harms among female sex workers following HIV self-test distribution in Malawi: results of a cohort study

**DOI:** 10.1186/s12879-024-09178-3

**Published:** 2024-03-11

**Authors:** Paul Mee, Melissa Neuman, Moses Kumwenda, Wezzie S. Lora, Simon Sikwese, Mwiza Sambo, Katherine Fielding, Pitchaya P. Indravudh, Karin Hatzold, Cheryl Johnson, Elizabeth. L. Corbett, Nicola Desmond

**Affiliations:** 1 Lincoln International Institute for Rural Health, College of Health and Science, University of Lincoln, Lincoln, UK; 2 Faculty of Epidemiology and Population Health, London School of Hygiene and Tropical Medicine, London, UK; 3 MRC International Statistics and Epidemiology Group, London School of Hygiene and Tropical Medicine, London, UK; 4 Malawi-Liverpool Wellcome Trust Clinical Research Programme, Blantyre, Malawi; 5 Pakachere Institute of Health and Development Communication, Blantyre, Malawi; 6 Faculty of Public Health and Policy, London School of Hygiene and Tropical Medicine, London, UK; 7 Population Services International, Cape Town, South Africa; 8 Global HIV, Hepatitis, STI Programmes, World Health Organization, Geneva, Switzerland; 9 Present Address: Department of International Public Health, Liverpool School of Tropical Medicine, Liverpool, UK

**Keywords:** HIV self-test, Female Sex Worker, Social harm, Malawi, Sub-Saharan Africa, Coercion, Intimate Partner Violence

## Abstract

**Background:**

In Malawi, female sex workers (FSW) have high HIV incidence and regular testing is suggested. HIV self-testing (HIVST) is a safe and acceptable alternative to standard testing services. This study assessed; whether social harms were more likely to be reported after HIVST distribution to FSW by peer distributors than after facility-based HIV testing and whether FSW regretted HIVST use or experienced associated relationship problems.

**Methods:**

Peer HIVST distributors, who were FSW, were recruited in Blantyre district, Malawi between February and July 2017. Among HIVST recipients a prospective cohort was recruited. Interviews were conducted at baseline and at end-line, 3 months later. Participants completed daily sexual activity diaries. End-line data were analysed using logistic regression to assess whether regret or relationship problems were associated with HIVST use. Sexual activity data were analysed using Generalised Estimating Equations to assess whether HIVST use was temporally associated with an increase in social harms.

**Results:**

Of 265 FSW recruited and offered HIVST, 131 completed both interviews. Of these, 31/131(23.7%) reported initial regret after HIVST use, this reduced to 23/131(17.6%) at the 3-month follow-up. Relationship problems were reported by 12/131(9.2%). Regret about HIVST use was less commonly reported in those aged 26–35 years compared to those aged 16–25 years (OR immediate regret—0.40 95% CI 0.16–1.01) (OR current regret—0.22 95% CI 0.07 – 0.71) and was not associated with the HIVST result. There was limited evidence that reports of verbal abuse perpetrated by clients in the week following HIVST use were greater than when there was no testing in the preceding week. There was no evidence for increases in any other social harms. There was some evidence of coercion to test, most commonly initiated by the peer distributor.

**Conclusions:**

Little evidence was found that the peer distribution model was associated with increased levels of social harms, however programmes aimed at reaching FSW need to carefully consider possible unintended consequences of their service delivery approaches, including the potential for peer distributors to coerce individuals to test or disclose their test results and alternative distribution models may need to be considered.

**Supplementary Information:**

The online version contains supplementary material available at 10.1186/s12879-024-09178-3.

## Background

In Malawi adult HIV prevalence remains high, with pronounced social and economic inequity in access to HIV prevention, testing and care services. HIV prevalence is even higher among key populations, specifically female sex workers (FSW) due to their high risk of HIV infection [1]. FSW also experience barriers in accessing HIV testing and treatment, due in part to the high level of stigma that they experience when accessing healthcare [2]. These barriers include factors such as their high levels of mobility, difficulties in accessing facility-based services during normal operating hours, and concerns about criminalization and stigmatised reactions from health workers [3]. Due to these issues, HIV testing services (HTS) need to be adapted to improve testing coverage and frequency among FSW in Malawi. With appropriate provision of care following HIV testing, high levels of engagement and retention are achievable for FSW [4, 5]. Additionally, and particularly in low and middle-income countries (LMIC), FSW experience high levels of sexual and physical violence. According to a global systematic review, 45–75% of sex workers reported at least one event of sexual or physical violence in their lifetime – with 32–55% reporting at least one event in the past year [6].

Globally 15% of the HIV burden among females has been shown to be directly attributable to participation in sex work, considering the potential for onward infection in partners and clients of FSW, the population level burden is substantially higher [7]. Thus increasing the engagement of FSW with HIV testing treatment and prevention services has the potential to have a significant impact on the trajectory of the HIV pandemic, particularly in resource-poor regions of Sub-Saharan Africa [5].

Whilst the knowledge of HIV status has increased across sub-Saharan Africa over time, the yield of positive tests and first-time diagnoses has dropped, indicating the need for alternative testing strategies [8]. HIV self-testing (HIVST) has been shown in a variety of settings in sub-Saharan Africa (SSA) to be a safe and acceptable alternative to health facility based testing [9–11].

Randomised controlled trials have shown that the provision of HIVST to FSW can lead to an increased uptake and frequency of testing [5, 12, 13] with different distribution methods being preferred depending on the local context [5]. The distribution of HIVST kits among peers has been shown to work effectively in studies working with FSW in Malawi, Zimbabwe, Zambia and Uganda [5, 14, 15]. The use of HIVST has also been shown to increase levels of testing among FSW, although the yield of positive test results remained similar to that for health facility based testing [16]. It has also been shown that FSW can act as effective distributors of HIVST to partners [17]. Further it has been found that the use of HIVST does not lead to an increase in sexual risk-taking among FSW [18] with evidence for reductions in some high risk sexual behaviour [19]. Concerns have been expressed about the risk of social harms and coercive testing associated with the use of HIVST by FSW [20, 21]. Two studies of HIVST use among FSW showed higher reported levels of social harm in the HIVST arm compared to those using health facility based HIV testing, though in each case the differences between the two arms were not statistically significant [13, 14].

In this study, we analysed the levels of expressed regret about testing following the use of HIVST by FSW and whether participants reported that they had experienced problems in the relationships with their partners that were caused by the self-test. We also assessed whether social harms were more likely to be experienced in the week following HIVST use than after health facility-based HIV testing. The research was a component of a programme of studies into HIVST distribution in Malawi [20] and nested within the wider programme of studies within the Unitaid/PSI HIV Self-Testing Africa (STAR) Initiative, the largest evaluation of HIV self-testing (HIVST) in Africa to date (https://hivstar.lshtm.ac.uk/**).**

## Methods

### Study design

This was a quantitative analysis using data from a mixed-methods study monitoring social harms among FSW as part of a pilot distribution of HIVST [21]. The OraQuick HIV self-test (Orasure Technologies LLC, Bethlehem, PA) was used with WHO pre-qualification approval.

Following a rapid ethnographic assessment and situational analysis, participatory development workshops were held to optimise delivery approaches for HIVST. Peer distributors were identified by a purposive method following participant observation in selected bars and sex-worker locations in order to identify those who would be most suitable for the role [5]. This was facilitated by the Pakachere Institute of Health and Development Communication, a non-governmental organisation based in Blantyre, Malawi. Those recruited were trained to act as HIVST distributors and to provide support on how to self-test and link to further HIV testing, treatment, and prevention services.

FSW were then recruited by peer distributors and initially given two HIVST kits [21, 22]. They could obtain additional kits from the peer-distributor at any time during the follow-up period. We did not monitor who used the test kits other than use by FSW. Since FSW were the focus of the research the ‘harm’ was reported harm by FSW. The peer distributors were trained to enable them to provide onward training on how to use and interpret the HIVST. To support the distribution process, non-cash incentives, such as scarves, were given to the peer distributors who distributed the highest number of kits, and all peer distributors received a monthly honorarium. A prospective cohort was recruited from the HIVST recipients between February and July 2017 distribution of HIVST kits also occurred over this period.

Interviews were conducted at baseline and three months later with FSW who accepted the HIVST kits using Audio-Computer Assisted Self-Interview (ACASI), in which participants were given a laptop or tablet computer and listened to pre-recorded questions to which they responded by selecting answers on a touch-screen or key pad [23] (see appendix for the interview questionnaire). Use of ACASI has been shown to reduce social-desirability bias when questions are asked relating to sensitive or stigmatised topics [24, 25]. At baseline, data were collected on individual socio-demographics, sexual behaviour, history of HIV testing and experience of social harms. Additionally, the 3-month follow-up interview included questions on coerced HIVST use or results disclosure, whether the respondent had experienced social harms, specifically either verbal abuse, physical abuse, sexual abuse, or the denial of economic resources from their stable partners. They were also asked whether they had regrets about using the HIVST, either at the time of testing or by the time of the 3-month follow-up interview or whether they had experienced relationship problems resulting from self-testing.

The details of the questions asked, translated into English, were:

- Immediate regret – “Aside from the results, did you have any regrets about your self-test immediately after you completed the test?”
- Current regret – “Looking back on your self-test now, do you regret taking this test now?”
- Relationship problems – “Were there any problems in your relationship caused by your self-test?”
- Verbal abuse – “In the past 3 months did your partner do the following to you? Insulted you; made you feel bad; belittled, humiliated, scared you (yelled or smashed things), or threatened to hurt you?”
- Physical abuse – “In the past 3 months did your partner do the following to you? Slapped, pushed, shoved, hit you with a fist, kicked, dragged, beaten you, choked, burned you, or threatened to use a gun, knife, or other weapon against you?”
- Sexual abuse – “In the past 3 months did your partner do the following to you? Forced you to have sexual intercourse by holding you down or making you afraid of him or forced you to do something sexual that you found humiliating?”
- Denial of economic resources – “In the past 3 months did your partner keep you from having the money you needed to buy food or other necessities even when he had money for other things?”

**Fig. 1:**
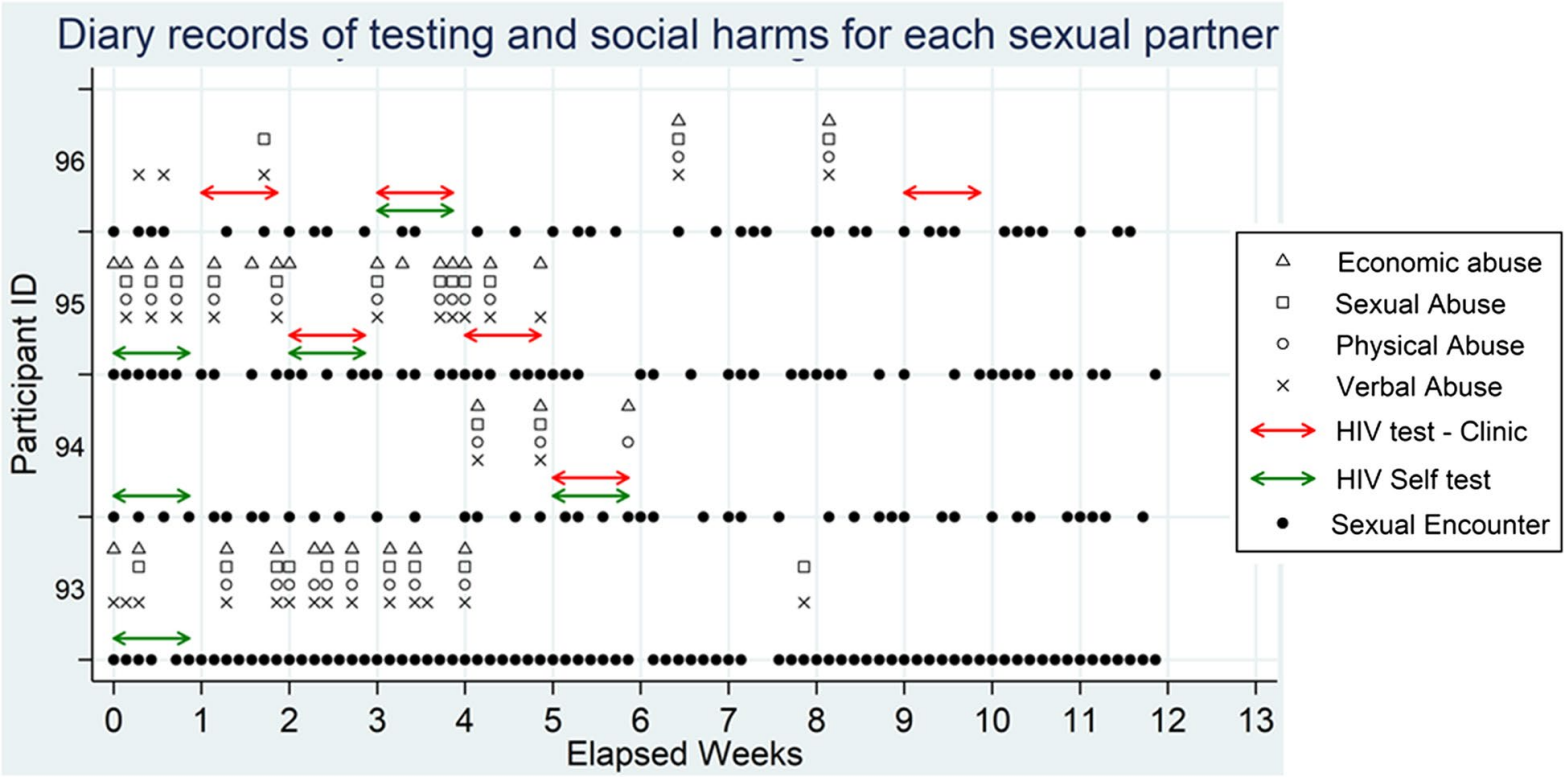
Graphical representation illustrating data recorded by female sex workers in their longitudinal diary. The black dots represent the occurrence of one or more sexual encounters, the other symbols indicate whether different forms of abuse were experienced on a particular day. The red and green arrows indicate that an HIV test was carried out at some point in the preceding week, the type of test being indicated by the colour of the arrow

Participants were also asked to complete a longitudinal sexual behaviour and social harms diary (Additional File 2—Social harms diary) in which the quantity and nature of sexual behaviours and their experiences of social harms were recorded each day. These included reports on all sexual acts with either clients or stable partners and whether they experienced verbal, physical or sexual abuse or the denial of economic resources from that partner on that day. HIV testing was recorded weekly over the 12-week period between the baseline and end-line interviews. One diary booklet contained 14 pages, covering a two-week period, and was collected for data entry every two weeks. Figure 1 presents a graphical representation of the data collected in the diary.

**Fig. 2:**
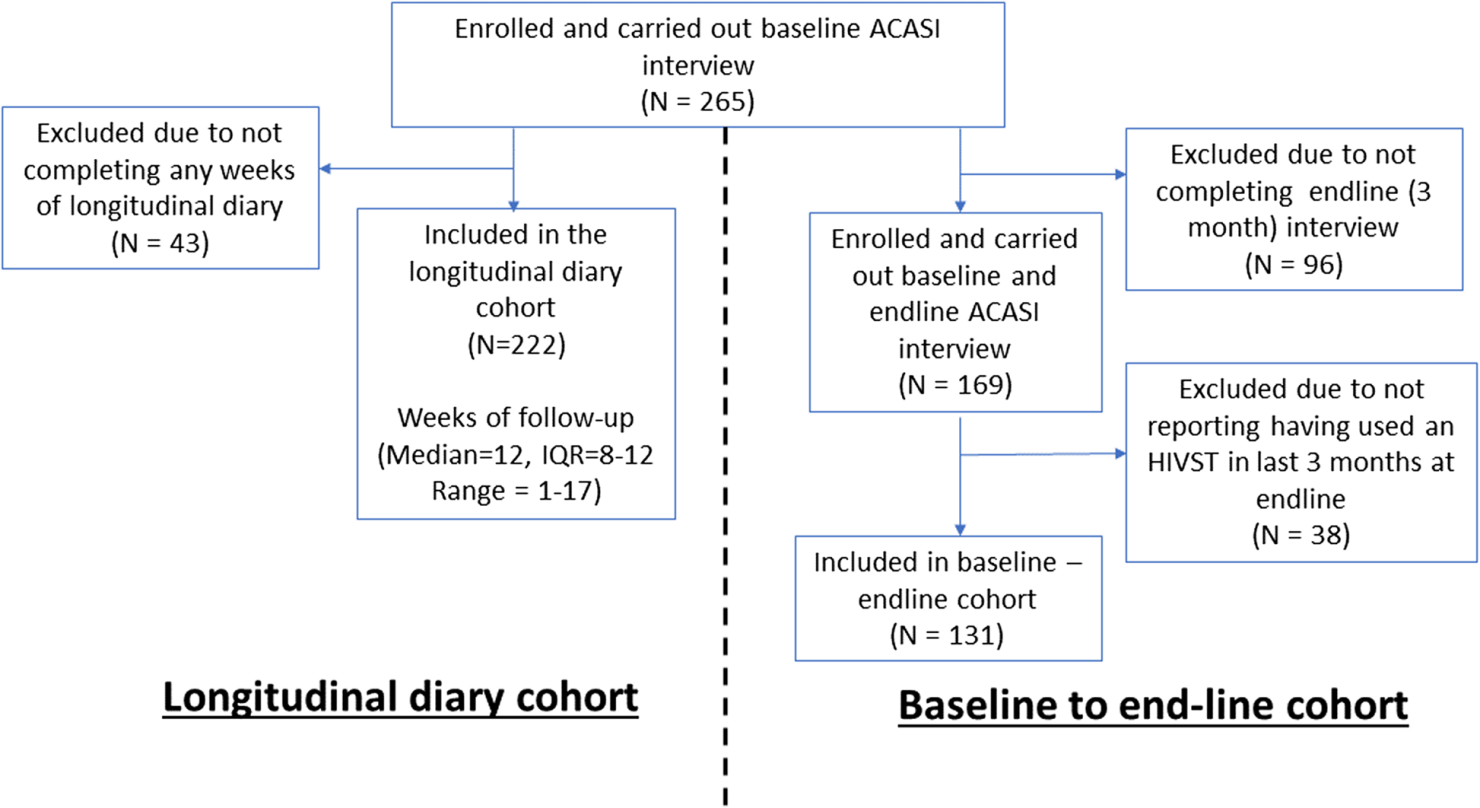
Study flow chart (Data were analysed from two cohorts derived from the initial population of all those who had completed a baseline ACASI interview.) showing reasons for exclusions

Two cohorts were constructed from the enrolled participants. The baseline to end-line cohort comprised all those who had completed both ACASI interviews and reported at the second interview that they had used an HIVST kit in the previous 3 months. The longitudinal diary cohort included all those who completed at least one week’s data in a sexual behaviour diary. (Fig. 2).

### Statistical analysis

Using the baseline to end-line cohort we analysed factors associated with outcomes of: (i) immediate regret at the time of HIVST use; (ii) current regret; and (iii) relationship problems associated with self-testing over the three months period following a self-test. All outcomes were binary (yes/no) and measured at the end-line (3-month) interview. Explanatory variables included were: the individual who initiated the self-test process (self or other), HIVST result (reactive/unreactive), the age and highest level of educational attainment (primary or lower/secondary or higher) of the participant.

Univariable logistic regression analyses were conducted to test whether there was evidence for an association between individual covariates and the outcomes. We also analysed whether there was evidence for the statistical confounding of any of these associations.

Using the longitudinal sexual behaviour diaries cohort, we assessed whether HIV testing behaviour in the previous week defined as either: no test, facility-based test only, self-test only or facility-based test and self-test, was followed by an increased risk of reporting social harms (verbal, physical or sexual abuse or the denial of economic resources) in the current week. Each type of social harm, recorded daily, was aggregated to a weekly measure defined as any occurrence in that week. Other covariates included in this analysis were: age, educational attainment, the number of reported sexual encounters in the current week and whether the individual had received material rewards in exchange for sex. Repeated weekly data per individual were analysed using generalized estimating equations (GEE) with a logit link function and an exchangeable autocorrelation structure. This method enabled an assessment of whether the outcome, occurrence of social harms, was statistically correlated with HIV testing, whilst taking into account the temporal correlation of events within individuals. Analyses were conducted separately for each type of social harm.

Covariates which showed statistical evidence for an association with the outcome in the univariable analyses were included in multivariable regression models. In a secondary analysis we limited the dataset to only those reports of social harm perpetrated by clients of the FSW to assess whether the results differed according to the type of sexual partner.

In each of the regression analyses a complete case analysis approach was used with only the cases in the data set for which there were no missing values on any of the variables included. All statistical analyses were carried out using the Stata statistical software v14.2 [26].

## Results

### Recruitment into the study

265 individuals were recruited into the study, all of whom accepted two HIVST kits from a peer distributor and completed a baseline ACASI interview. At the three-month follow-up, 169 completed an ACASI interview (Fig. 2). A further 38 were excluded as they reported not having self-tested in last three months. A total of 131 individuals were included in the baseline to end-line cohort.

Those excluded from the baseline to end-line cohort tended to be younger than those included, 63.4% (*n* = 85) of those excluded were in the age range 16 to 25 compared to 50.4% (*n* = 66) of those included (Chi-squared p-value comparing the distributions of the population by age = 0.07). There was no statistical evidence for a difference in the distributions of marital status or educational attainment level between the two groups (Additional file 1, Table S1). In the longitudinal diary cohort, 43 individuals were excluded as none of their weekly sexual encounter diaries were captured, leaving a total of 222 individuals (Additional file 1, Table S2). Almost all 130/131 of the individuals in the baseline-endline cohort were included in the longitudinal diary cohort. There was no statistical evidence that the distributions of the characteristics of individuals differed between the two cohorts (Additional file 1, Table S2).

### Analysis of the baseline to end-line interview data

At the three-month follow-up interview, 23.7% (31/131) of the participants reported that they had regretted using HIVST at the time of testing. This figure decreased to 17.6% (23/131) reporting that they still felt regret at the time of the interview. Of the cohort 9.2% (12/131) reported that they had experienced relationship problems associated with using the HIVST (Table 1). Out of 104 individuals responding, 44 stated that someone else had initiated the use of the HIVST, in 39 cases this person was the peer distributor. There was no evidence that the levels of regret or relationship problems were greater if another individual initiated HIVST use.

**Table 1:** Factors associated with regret/relationship problems as reported at the end-line (3 month) interview

Variable	Category	Immediate regret about HIVST use (reported at 3 months)^a^			Current regret about HIVST use (reported at 3 months)^a^			Relationship problems associated with HIVST use (reported at 3 months)^a^		
		**n/N (%)**^**a**^	**OR (95% CI)**	***p*-value**	**n/N (%)**^**a**^	**OR (95% CI)**	***p*-value**	**n/N (%)**^**a**^	**OR (95% CI)**	***p*-value**
**Initiator of HIVST**	**Self**	20/60(33.3)	1	0.36	14/60(23.3)	1	0.73	4/60(6.7)	1	0.12
	**Other**	11/44(25.0)	0.67(0.28—1.59)		9/44(20.5)	0.84(0.33—2.18)		8/44(18.2)	2.83(0.77—10.49)	
**HIVST result**	**Unreactive**	21/67(31.3)	1	0.65	12/67(17.9)	1	0.17	8/67(11.9)	1	0.69
	**Reactive**	10/37(27.0)	0.81(0.33—1.98)		11/37(29.7)	1.94(0.76—4.97)		4/37(10.8)	1.31(0.35—4.97)	
**Age range (years)**	**16–25**	21/66(31.8)	1	0.06	17/66(25.8)	1	0.04	7/66(10.6)	1	0.87
	**26–35**	9/53(17.0)	0.40(0.16—1.01)		4/53(7.5)	0.22(0.07—0.71)		4/53(7.5)	0.72(0.19—2.76)	
	** > = 36**	1/12(8.3)	0.16(0.02—1.39)		2/12(16.7)	0.51(0.10—2.69)		1/12(8.3)	0.69(0.07—6.70)	
**Maximum level of educational attainment**	**Primary or less**	23/82(28.0)	1	0.18	16/82(19.5)	1	0.56	9/82(11.0)	1	0.15
	**Secondary or higher**	8/49(16.3)	0.53(0.21—1.34)		7/49(14.3)	0.74(0.27—2.01)		3/49(6.1)	0.36(0.09—1.45)	
**Total n/N (%)**		**31/131 (23.7)**			**23/131 (17.6)**			**12/131 (9.2)**		

^a^Missing responses were not included in the table, 27/131 (20.6%) of respondents had missing responses to the questions on whether they experienced immediate or current regret about the use of HIVST and 61/131 (46.6%) of respondents had missing responses to the question on whether they experienced relationship problems associated with the use of HIVST. There was no missing data for the other covariates and no invalid or uncertain HIVST test results reported

There was some evidence that the proportions of women expressing immediate or current regret was lower for those of older age (Table 1). For those expressing immediate regret the odds ratio (OR) for those aged 26 to 35 compared to those aged 16 to 25 was 0.40 (95%CI:0.16–1.01). For current regret comparing the same age groups the OR was 0.22 (95%CI:0.07–0.71). There was a high level of non-response: 27/131 (20.6%) had missing data on immediate or current regret and 61/131 (46.6%) had missing data on experiences of relationship problems following the use of the HIVST.

The forced use of the HIVST kit was reported by 10/131 (7.6%) of the participants and forced disclosure of the results by 11/131 (8.4%) (Table 2). There was some evidence that participants were more likely to have been forced to test (OR 15.17 -95%CI:1.84–124.87) or forced to disclose their test results (OR 4.22—95%CI:1.05—16.97) if the test was initiated by someone other than themselves.

**Table 2:** Factors associated with regret/relationship problems as reported at the end-line (3 month) interview

Variable	Category	Forced to use HIVST (Reported at 3 months)^a^			Forced to disclose results of HIVST (Reported at 3 months)^a^		
		**n/N (%)**^**a**^	**OR (95% CI)**	***p*-value**	**n/N (%)**^**a**^	**OR (95% CI)**	***p*-value**
**Initiator of HIVST**	**Self**	1/60(1.7)	1	0.01	3/60(5.0)	1	0.04
	**Other**	9/44(20.5)	15.17(1.84—124.87)		8/44(18.2)	4.22(1.05—16.97)	
**HIVST result**	**Unreactive**	4/67(6.0)	1	0.10	6/67(9.0)	1	0.47
	**Reactive**	6/37(16.2)	3.05(0.80—11.60)		5/37(13.5)	1.59(0.45—5.61)	
**Age range (years)**	**16–25**	3/66(4.5)	1	0.33	6/66(9.1)	1	0.49
	**26–35**	5/53(9.4)	2.21(0.50—9.83)		3/53(5.7)	0.59(0.14—2.51)	
	** > = 36**	2/12(16.7)	4.08(0.59—28.39)		2/12(16.7)	1.92(0.33—11.23)	
**Maximum levels of educational attainment**	**Primary or less**	8/82(9.8)	1	0.29	8/82(9.8)	1	0.55
	**Secondary or higher**	2/49(4.1)	0.42(0.09—2.10)		3/49(6.1)	0.65(0.16—2.62)	
**Total n/N (%)**		**10/131(7.6)**			**11/131(8.4)**		

^a^Missing responses were not included in the table, 27/131 (20.6%) of respondents had missing responses to the question on forced testing and 27/31 (20.6%) had missing responses to the question on whether they had been forced to disclose their test results. There was no missing data for the other covariates and no invalid or uncertain HIVST test results reported

To test whether age was a confounder we developed contingency tables and calculated Chi-squared p-values for associations between age group and test initiator, HIVST result and maximum educational attainment (Additional file 1, Tables S3, S4, S5). There was no evidence from these of any associations between the pairs of variables and for this reason we did not carry out multivariable analyses.

### Analysis of the associations between HIV testing in the previous week and the occurrence of social harms in the current week

A total of 222 women contributed 1257 weeks of follow-up data (median 12 weeks, range 1–17) to this analysis (Fig. 2). A total of 29,194 sexual encounters were reported, of these 24,123 (82.6%) were with a client and 5071 (17.4%) with a stable partner. There was no statistical evidence from this pooled dataset that either facility-based HIV testing or HIVST in the previous week resulted in increased reports of any form of social harm in the current week. (Tables 3, 4, 5 and 6).

**Table 3:** The association between HIV testing in the previous week and verbal abuse in the current week, using GEE

Variable	Category	Reports of verbal abuse (reports/total weeks reported) (%)^a^	Univariable analysis OR (95% CI)	*p*-value	Multivariable analysis OR (95% CI)^b^	*p*-value
**Test type in previous week**	**No test**	148/1083 (13.7)	1	0.83	1	0.50
	**Clinic-based test**	7/47 (14.9)	1.28(0.63—2.61)		1.22(0.57—2.59)	
	**Self-test**	18/100 (18.0)	1.23(0.60—2.52)		1.46(0.87—2.47)	
	**Clinic test & Self-test**	2/27 (7.4)	0.80(0.24—2.65)		0.83(0.27—2.58)	
**Sexual encounters in the current week**	**0–9**	62/593 (10.5)	1	< 0.01	1	< 0.01
	**10–20**	84/399 (21.1)	2.29(1.53—3.44)		2.32(1.55—3.49)	
	** > 20**	29/265 (10.9)	2.19(1.28—3.74)		2.21(1.29—3.79)	
**Age range (years)**	**16—25**	105/621 (16.9)	1	0.28		
	**26—35**	59/512 (11.5)	0.63 (0.33–1.21)			
	** > = 36**	11/124 (8.9)	0.52 (0.15–1.74)			
**Educational Attainment**	**Primary or less**	119/826 (14.4)	1	0.58		
	**Secondary or higher**	56/431 (13.0)	0.84 (0.44–1.59)			
**Received material goods or payment in exchange for sex**	**No**	53/405 (13.1)	1	0.70		
	**Yes**	101/651 (15.5)	1.14 (0.58–2.27)			
	**Missing**	21/201(10.4)				

^a^A total of 175 reports of verbal abuse were recorded in 1257 weeks of data collection

^b^Only variables showing an association with the outcome in univariable analyses were included in the multivariable model in which odds ratios were adjusted test-type in the previous week and number of sexual encounters in the current week

**Table 4:** The association between HIV testing in the previous week and physical abuse in the current week, using GEE

Variable	Category	Reports of physical abuse (reports/total weeks reported) (%)^a^	Univariable analysis OR (95% CI)	*p*-value	Multivariable analysis OR (95% CI)^b^	*p*-value
**Test type in previous week**	**No test**	136/1083 (12.6)	1	0.34	1	0.57
	**Clinic-based test**	4/47 (8.5)	0.92(0.42—2.00)		0.70(0.28—1.71)	
	**Self-test**	15/100 (15.0)	1.67(0.88—3.19)		1.29(0.76—2.18)	
	**Clinic test & Self-test**	1/27 (3.7)	0.51(0.12—2.12)		0.66(0.20—2.19)	
**Sexual encounters in the current week**	**0–9**	50/593 (8.4)	1	< 0.01	1	< 0.01
	**10–20**	81/399 (20.3)	2.56(1.68—3.90)		2.56(1.68—3.92)	
	** > 20**	25/265 (9.4)	2.50(1.43—4.36)		2.51(1.44—4.39)	
**Age range (years)**	**16—25**	90/621 (14.5)	1	0.41		
	**26—35**	60/512 (11.7)	0.78(0.37—1.63)			
	** > = 36**	6/124 (4.8)	0.31(0.05—1.91)			
**Educational Attainment**	**Primary or less**	111/826 (13.4)	1	0.34		
	**Secondary or higher**	45/431 (10.4)	0.68(0.31—1.49)			
**Received material goods or payment in exchange for sex**	**No**	51/405 (12.6)	1	0.79		
	**Yes**	82/651 (12.6)	0.89(0.39—2.05)			
	**Missing**	23/201(11.4)				

^a^A total of 156 reports of physical abuse were recorded in 1257 weeks of data collection

^b^Only variables showing an association with the outcome in univariable analyses were included in the multivariable model in which odds ratios were adjusted test-type in the previous week and number of sexual encounters in the current week

**Table 5:** The association between HIV testing in the previous week and sexual abuse in the current week, using GEE

Variable	Category	Reports of sexual abuse (reports/total weeks reported) (%)^a^	Univariable analysis OR (95% CI)	*p*-value	Multivariable analysis OR (95% CI)^b^	*p*-value
**Test type in previous week**	**No test**	131/1083 (12.1)	1	0.59	1	0.70
	**Clinic-based test**	6/47 (12.8)	1.19(0.56—2.52)		0.95(0.41—2.20)	
	**Self-test**	13/100 (13.0)	1.42(0.70—2.88)		1.22(0.69—2.14)	
	**Clinic test & Self-test**	1/27 (3.7)	0.51(0.11—2.28)		0.48(0.11—2.11)	
**Sexual encounters in the current week**	**0–9**	51/593 (8.6)	1	< 0.01	1	< 0.01
	**10–20**	77/399 (19.3)	2.29(1.49—3.50)		2.29 (1.49—3.50)	
	** > 20**	23/265 (8.7)	1.97(1.11—3.50)		1.98 (1.11—3.52)	
**Age range (years)**	**16—25**	95/621 (15.3)	1	0.19		
	**26—35**	49/512 (9.6)	0.59(0.29—1.20)			
	** > = 36**	7/124 (5.6)	0.36(0.08—1.68)			
**Educational Attainment**	**Primary or less**	102/826 (12.3)	1	0.63		
	**Secondary or higher**	49/431 (11.4)	0.84(0.40—1.73)			
**Received material goods or payment in exchange for sex**	**No**	50/405 (12.3)	1	0.87		
	**Yes**	87/651 (13.4)	1.06(0.50—2.28)			
	**Missing**	14/201(7.0)				

^a^A total of 175 reports of sexual abuse were recorded in 1257 weeks of data collection, missing data for covariate categories was not included in the table

^b^Only variables showing an association with the outcome in univariable analyses were included in the multivariable model in which odds ratios were adjusted test-type in the previous week and number of sexual encounters in the current week

**Table 6:** The association between HIV testing in the previous week and economic abuse (denial of economic resources) in the current week, using GEE

Variable	Category	Reports of economic abuse (reports/total weeks reported) (%)^a^	Univariable analysis OR (95% CI)	*p*-value	Multivariable analysis OR (95% CI)^b^	*p*-value
**Test type in previous week**	**No test**	184/1083 (17.0)	1	0.24	1	0.35
	**Clinic-based test**	8/47 (17.0)	1.15(0.63—2.10)		1.12(0.61—2.06)	
	**Self-test**	17/100 (17.0)	1.36(0.76—2.42)		1.29(0.84—1.99)	
	**Clinic test & Self-test**	1/27 (3.7)	1.94(0.92—4.06)		0.47(0.15—1.44)	
**Sexual encounters in the current week**	**0–9**	84/593 (14.2)	1	0.02	1	0.02
	**10–20**	98/399 (24.6)	1.52(1.10—2.10)		1.52(1.10—2.10)	
	** > 20**	28/265 (10.6)	1.70(1.08—2.66)		1.70(1.09—2.67)	
**Age range (years)**	**16—25**	116/621 (18.7)	1	0.56		
	**26—35**	82/512 (16.0)	0.79(0.38—1.62)			
	** > = 36**	12/124 (9.7)	0.48(0.11—2.09)			
**Educational Attainment**	**Primary or less**	129/826 (15.6)	1	0.60		
	**Secondary or higher**	81/431 (18.8)	1.21(0.59—2.45)			
**Received material goods or payment in exchange for sex**	**No**	90/405 (22.2)	1	0.15		
	**Yes**	94/651 (14.4)	0.57(0.27—1.23)			
	**Missing**	26/201(12.9)				

^a^A total of 210 reports of economic abuse were recorded in 1257 weeks of data collection, missing data for covariate categories was not included in the table

^b^Only variables showing an association with the outcome in univariable analyses were included in the multivariable model in which odds ratios were adjusted test-type in the previous week and number of sexual encounters in the current week

There was strong evidence that the odds of experiencing social harms increased as the number of sexual encounters in the current week increased, this was true for all forms of social harm. The ORs for reporting verbal, physical, sexual abuse or denial of economic resources perpetrated by either clients or regular partners for those reporting 10 to 20 sexual encounters compared to those reporting 0 to 9 encounters were 2.29 (95%CI:1.53–3.45), 2.56 (95%CI:1.68–3.90), 2.29 (95%CI: 1.49–3.50) and 1.52 (95%CI:1.10–2.10), respectively. There was no evidence that the reports of social harms differed by age, educational attainment or whether the participant self-reported that they engaged in sexual activity in exchange for material or financial payment.

A further analysis was carried out to investigate whether there was evidence that the findings differed according to whether the sexual partner was a stable partner or a client **(**Additional file 1, Tables S6, S7, S8, S9). In this we limited the analytical dataset to include only those sexual encounters occurring with clients. After adjusting for the number of sexual encounters reported in the current week there was some evidence that the odds of a woman experiencing verbal abuse from clients were greater if HIV self-testing occurred in the previous week than if no testing had occurred in the previous week (OR 1.91 (95% CI:1.12–3.27). There was no evidence for an increase in the occurrence of any other types of social harm perpetrated by clients following HIV testing.

## Discussion

This study aimed to assess whether there was any evidence that additional social harms were associated with the use of HIVST amongst FSW in this study setting through a longitudinal approach situating occurrences of harms temporally with the use of HIVST. It also aimed to contribute to our understanding of the acceptability of HIVST amongst FSW through the analysis of reports of regret and relationship problems following HIVST use. The findings are particularly relevant to inform strategies for the scale-up of HIVST use for FSW in contexts with high background rates of gender-based violence (GBV) such as Malawi where 41% of women report ever having experienced an episode of sexual or physical violence [27].

This study found no evidence in the pooled data on all sexual activity that social harms were more likely to occur in the week following an HIVST. There was however some evidence, that participants experienced increased levels of verbal abuse from their clients in the week following the use of an HIVST. Expressions of regret about the use of the HIVST were common. Among the participants 23.7% expressed feelings of regret at the time they used the HIVST, decreasing to 17.6% after three months. Relationship problems after using HIVST were reported by 9.2% of participants.

Whilst this quantitative study did not explore the reasons for expressions of regret by female sex workers, separate qualitative research involving participants from the same population explored these factors [20]. Some participants in that study expressed regret at testing due to being forced, most commonly by the peer distributor, to either carry out the test or disclose test results.

Some women experienced violence, mostly perpetrated by an established partner and linked to disclosure of results or following their request that a partner also test. However, feelings of regret expressed by some women did not translate to an unwillingness to test again through HIVST in the future [5, 20].

It was not possible from the data collected to compare this with the level of reported relationship problems amongst those not using an HIVST. We did not monitor who used the test kits other than use by FSW—since FSW were the focus of the research the ‘harm’ was reported harm by FSW. A qualitative study accompanying this reported that participants expressed “excitement” about the peer HIVST distribution, indicating that they felt it had several advantages over clinic-based testing as it provided greater confidentiality, was more flexible, less costly, easier to use and less intrusive for those in high HIV-risk occupations [5]

One concern about the use of HIVST is the potential for an individual to be coerced by someone else into taking the test, thus violating their autonomy. Previous studies in the general population have shown that where there is pressure to use an HIVST it is most likely to come from an individual’s partner [28]. It has also been shown that among cohabiting couples in Malawi, pressure to test was usually considered well-intentioned on the part of the person persuading their partner to test [20]. In the model used in this study, the peer distributors were trained to support the use of the HIVST by the participants, thus they may have been more involved in the testing process than would be the case in other self-testing models. It is possible that the non-financial incentives given to the most successful peer distributors may have led to greater levels of coercive testing than would otherwise have occurred. Evidence from qualitative interviews with the participants indicated that venue-based FSW were more vulnerable to coercive testing as they were dependent on the venue owner for their opportunity to work and it was found that in some cases the peer distributors took advantage of this situation [21]. This may explain why over 30% of the individuals with an unreactive HIVST result reported that they immediately regretted their use of the test. The finding of marginally increased levels of reported verbal abuse from clients in the week following an HIVST compared to weeks in which no testing occurred, was unexpected and needs further investigation. It may indicate that there was a breakdown in the confidentiality and privacy which should be associated with HIVST use in the distribution model used in this study.

A previous study comparing different HIVST distribution strategies for FSW in Zimbabwe and Malawi [5] indicated that where there was a strong existing HIV testing programme for FSW, as was the case in Zimbabwe, there was a preference for facility-based testing. Where such provision was not in place, as in Malawi, a peer distribution strategy was found to be preferable. Due to this and concerns around the potential for coerced testing in the peer distribution model [5], it is important that alternatives approaches to distribute HIVST to FSW are considered in different settings. Examples of possible alternative models include the direct provision of an HIVST from a healthcare worker or the provision of a coupon allowing individuals to collect an HIVST from a health facility [18].

There have also been concerns expressed that individuals using an HIVST will not have appropriate counselling and support in the event that they receive a reactive (positive) test result, although there is little evidence to support this concern [28]. The fact that in this study there was no evidence that the expressions of regret were associated with the HIVST result suggests that the reasons underlying this feeling of regret are more nuanced. Further qualitative research is needed to understand in more depth the meaning behind an expression of “regret” in this cultural context.

A limitation of this study was that due to the temporal resolution of the data we could not investigate short term effects of HIV testing on social harms occurring in the same week as the test. Also, due to the relatively high levels of non-response to the questions about regret or relationship problems, these results may be biased. Out of 265 individuals initially enrolled, 134 were excluded from the baseline to end-line cohort and 43 from the longitudinal diary cohort. As a result, the study populations had a somewhat older age profile than all participants recruited but otherwise was representative of those recruited into the study. Among the strengths of the study were that as the baseline and end-line surveys were carried out using the ACASI system, in which data was entered directly by the participants rather than being reported verbally to a research team member, the accuracy of the responses to potentially sensitive information was likely to be greater. The use of daily longitudinal diaries to collect sexual behaviour data reduced the potential for recall bias and enabled a fine-grained temporal analysis of this data.

## Conclusion

Our study found little evidence that introducing HIVST amongst FSW in Malawi using a peer distribution model was associated with subsequent increased levels of social harms, however the use of peer distribution agent incentives and the potential for venue owners to coerce FSW to test may have compromised the freedom of choice of some FSW. It is possible that this may ultimately impact long term retention in care as participants react against what they perceive to be forced participation. This reinforces the importance of understanding in detail the local sex work environment when developing programmes designed to promote engagement with HIV services for FSW. Relatively high levels of regret about HIVST use were reported but this decreased over time, and these were not associated with the HIVST result. It should be emphasised that the limited evidence for statistical associations reported here do not imply a causal link between HIVST use and any form of social harm. Further qualitative studies are needed to understand the meaning of regret in this cultural context. Based on this and the accompanying qualitative evidence we can conclude that peer distribution of HIVST provides a preferred and safe alternative to facility-based testing for FSW in this setting. Future programmes need to prioritize working with FSWs and consider alternative strategies to peer distribution in order to design safe and effective HIVST service delivery approaches.

### Supplementary Information

**Additional file 1: Tables S1 - S9.**

**Additional file 2.** Social harms diary

**Additional file 3.** ACASI questionairre

## Data Availability

The datasets used and/or analysed during the current study are available from the corresponding author on reasonable request.

## Abbreviations

FSW: Female sex workers
HIV: Human Immunodeficiency virus
HIVST: HIV self-test
ACASI: Audio Computer Assisted Self-interviews
LMIC: Low- and middle-income countries
SSA: Sub-Saharan Africa
IPV: Intimate partner violence
OR: Odds ratio
GEE: Generalised Estimating Equations
CI: Confidence Interval
GBV: Gender-based violence
