# Supplementary material for: Experience of social harms among female sex workers following HIV self-test distribution in Malawi: results of a cohort study
Source: BMC Infect Dis. 2024 Mar 11;22(Suppl 1):978. doi: 10.1186/s12879-024-09178-3 (PMC10926537; doi:10.1186/s12879-024-09178-3)
Supplement: Supplementary file 1 — Additional file 1: Tables S1 - S9. [file 12879_2024_9178_MOESM1_ESM.pdf]

Additional File 1

Table S1 – Assessment of Data Quality – comparison of those included in and excluded from the baseline to end-line study cohort.

Variable	Category	Excluded from study cohort[1] n(%)	Included in study cohort n(%)	Total	Chi-squared p-value
**Age (years)**	16-25	85 (63.4)	66 (50.4)	151 (57.0)	0.07
	26-35	43 (32.1)	53 (40.5)	96 (36.2)	
	≥36	6 (4.5)	12 (9.2)	18 (6.8)	
**Highest level of education achieved**	Primary or less	88 (65.7)	82 (62.6)	170 (64.2)	0.61
	Secondary or higher	46 (34.3)	49 (37.4)	95 (35.8)	
**Marital status**	Married or living as married	39 (29.1)	38 (29.0)	77 (29.1)	0.64
	Never married	52 (38.8)	44 (33.6)	96 (36.2)	
	Widowed or separated	43 (32.1)	49 (37.4)	92 (34.7)	
	**Total**	**134**	**131**	**265**	

[1] 96 participants were excluded from the study cohort as they failed to complete the 3-month interview, a further 38 were excluded as they did not report having used an HIVST in the previous 3 months.

Table S2 – Comparison of the characteristics of those included in the baseline to end-line cohort and those included in the cohort for analysis of diary data.

Characteristic	Category	Diary cohort [1] n(%)	Baseline to end-line cohort [1] n(%)	Chi-squared p-value
**Age (years)**	**16-25**	118(53.2)	66(50.4)	0.29
	**26-35**	86(38.7)	53(40.5)	
	**>36**	18(8.1)	12(9.2)	
**Educational attainment**	**Primary or less**	142(64.0)	82(62.6)	0.80
	**Secondary or higher**	80(36.0)	49(37.4)	
**Marital status**	**Married or living as married**	65(29.3)	38(29.0)	1.00
	**Never married**	74(33.3)	44(33.6)	
	**Widowed/separated**	83(37.4)	49(37.4)	
**Previously tested for HIV**	**No**	29(13.1)	14(10.7)	0.51
	**Yes**	193(86.9)	117(89.3)	
**Received something in exchange for sex**	**No**	73(32.9)	44(33.6)	0.79
	**Yes**	115(51.8)	65(49.6)	
**Total**		**222**	**131**	

[1] Missing data was not included in the table.

There were 130 individuals were included in both cohorts with one participant exclusively in the baseline-endline cohort and 92 participants exclusively in the longitudinal diary cohort .A comparison of the characteristics between these two cohorts (Table S2) showed no evidence for a significant difference in the characteristics of the individuals included.

Table S3 - Contingency table showing the association between age group and initiator of HIV self-test.

		Age Group			
		**16-25 N (%)**	**26-35 N (%)**	**>= 36 N (%)**	**Total**
**Initiator of HIVST**	**Self**	26(43.3)	29(48.3)	5(8.3)	**60**
	**Other**	26(59.1)	13(29.5)	5(11.4)	**44**
	**Missing**	14(51.9)	11(40.7)	2(7.4)	**27**
	**Total**	**66(50.4)**	**53(40.5)**	**12(9.2)**	**131**
	**Chi-squared p-value**	**0.43**			

Table S4 - Contingency table showing the association between age group and HIV self-test result.

		Age Group			
		**16-25 N (%)**	**26-35 N (%)**	**>= 36 N (%)**	**Total**
**HIVST result**	**Unreactive**	39(58.2)	23(34.3)	5(7.5)	**67**
	**Reactive**	13(35.1)	19(51.4)	5(13.5)	**37**
	**Missing**	14(51.9)	11(40.7)	2(7.4)	**27**
	**Total**	**66(50.4)**	**53(40.5)**	**12(9.2)**	**131**
**Chi-squared**	**Chi-squared p-value**	**0.26**			

Table S5 - Contingency table showing the association between age group and maximum levels of educational attainment.

		Age Group			
		**16-25 N (%)**	**26-35 N (%)**	**>= 36 N (%)**	**Total**
**Maximum level of educational attainment**	**Primary or less**	40(48.8)	33(40.2)	9(11.0)	**82**
	**Secondary or higher**	26(53.1)	20(40.8)	3(6.1)	**49**
	**Total**	**66(50.4)**	**53(40.5)**	**12(9.2)**	**131**
	**Chi-squared p-value**	**0.64**			

Table S6 - The association between HIV testing in the previous week and verbal abuse in the current week perpetrated by clients, using GEE.

Variable	Category	Reports of verbal abuse (reports/total weeks reported) (%) [1]	Univariable analysis OR (95% CI)	p-value	Multivariable analysis OR (95% CI) [2]	p-value
Test type in previous week	No test	118/1013(11.6)	1	0.08	1	0.07
	Clinic test	4/43(9.3)	0.71 (0.25-1.97)		0.69 (0.25-1.95)	
	Self-test	17/99(17.2)	1.89 (1.12-3.21)		1.91 (1.12-3.27)	
	Clinic test & Self-test	1/27(3.7)	0.54 (0.12-2.51)		0.47 (0.09-2.46)	
Sexual encounters in the current week	0-9	80/672(11.9)	1	0.04	1	0.03
	10-20	47/290(16.2)	1.49 (0.98-2.26)		1.49 (0.98-2.25)	
	>20	13/220(5.9)	0.69 (0.33-1.43)		0.65 (0.31-1.35)	
Age range (years)	16 - 25	88/601(14.6)	1	0.28		
	26 - 35	44/469(9.4)	0.60 (0.30-1.23)			
	>= 36	8/112(7.1)	0.49 (0.13-1.89)			
Educational Attainment	Primary or less	101/791(12.8)	1	0.30		
	Secondary or higher	39/391(10.0)	0.68 (0.33-1.41)			
Received material goods or payment in exchange for sex	No	45/353(12.7)	1	0.85		
	Yes	76/633(12.0)	0.93 (0.43-2.00)			
	Missing	19/196(9.7)				

[1] **A total of 140 reports of verbal abuse were recorded in 1182 weeks of data collection.**

[2] **Only variables showing an association with the outcome in univariable analyses were included in the multivariable model.**

Table S7 - The association between HIV testing in the previous week and physical abuse in the current week perpetrated by clients, using GEE.

Variable	Category	Reports of physical abuse (reports/total weeks reported) (%) [1]	Univariable analysis OR (95% CI)	p-value	Multivariable analysis OR (95% CI) [2]	p-value
**Test type in previous week**	**No test**	113/1013(11.2)	1	0.26	1	0.26
	**Clinic test**	2/43(4.7)	0.32 (0.08-1.32)		0.32 (0.08-1.32)	
	**Self-test**	12/99(12.1)	1.12 (0.61-2.03)		1.12 (0.61-2.05)	
	**Clinic test & Self-test**	0/27(0.0)	-		-	
**Sexual encounters in the current week**	**0-9**	71/672(10.6)	1	0.01	1	0.01
	**10-20**	46/290(15.9)	1.76 (1.15-2.68)		1.76 (1.15-2.69)	
	**>20**	10/220(4.5)	0.94 (0.46-1.91)		0.90 (0.44-1.83)	
**Age range (years)**	**16 - 25**	75/601(12.5)	1	0.49		
	**26 - 35**	47/469(10.0)	0.78 (0.36-1.71)			
	**>= 36**	5/112(4.5)	0.35 (0.06-2.18)			
**Educational Attainment**	**Primary or less**	93/791(11.8)	1	0.29		
	**Secondary or higher**	34/391(8.7)	0.64 (0.28-1.48)			
**Received material goods or payment in exchange for sex**	**No**	41/353(11.6)	1	0.66		
	**Yes**	65/633(10.3)	0.89 (0.39-2.05)			
	**Missing**	21/196(10.7)				

[1] A total of 127 reports of physical abuse were recorded in 1182 weeks of data collection.

[2] Only variables showing an association with the outcome in univariable analyses were included in the multivariable model .

Table S8 - The association between HIV testing in the previous week and sexual abuse in the current week perpetrated by clients, using GEE.

Variable	Category	Reports of sexual abuse (reports/total weeks reported) (%) [1]	Univariable analysis OR (95% CI)	p-value	Multivariable analysis OR (95% CI) [2]	p-value
Test type in previous week	No test	104/1013(10.3)	1	0.29	1	0.26
	Clinic test	3/43(7.0)	0.47 (0.14-1.65)		0.46 (0.13-1.62)	
	Self-test	11/99(11.1)	1.35 (0.75-2.44)		1.38 (0.76-2.52)	
	Clinic test & Self-test	0/27(0.0)	-		-	
Sexual encounters in the current week	0-9	71/672(10.6)	1	0.01	1	0.01
	10-20	40/290(13.8)	1.50 (0.98-2.31)		1.49 (0.97-2.30)	
	>20	7/220(3.2)	0.41 (0.16-1.05)		0.39 (0.15-1.01)	
Age range (years)	16 - 25	78/601(13.0)	1	0.20		
	26 - 35	35/469(7.5)	0.54 (0.24-1.20)			
	>= 36	5/112(4.5)	0.37 (0.07-1.93)			
Educational Attainment	Primary or less	82/791(10.4)	1	0.57		
	Secondary or higher	36/391(9.2)	0.79 (0.35-1.78)			
Received material goods or payment in exchange for sex	No	38/353(10.8)	1	0.92		
	Yes	67/633(10.6)	0.96 (0.41-2.24)			
	Missing	13/196(6.6)				

[1] **A total of 118 reports of sexual abuse were recorded in 1182 weeks of data collection.**

[2] **Only variables showing an association with the outcome in univariable analyses were included in the multivariable model.**

Table S9 - The association between HIV testing in the previous week and economic abuse (denial of economic resources) in the current week perpetrated by clients, using GEE.

Variable	Category	Reports of economic abuse (reports/total weeks reported) (%) [1]	Univariable analysis OR (95% CI)	p-value	Multivariable analysis OR (95% CI) [2]	p-value
**Test type in previous week**	**No test**	142/1013(14.0)	1	0.19	1	0.17
	**Clinic test**	5/43(11.6)	0.87 (0.39-1.95)		0.88 (0.40-1.95)	
	**Self-test**	17/99(17.2)	1.61 (1.00-2.61)		1.63 (1.01-2.65)	
	**Clinic test & Self-test**	1/27(3.7)	0.59 (0.17-2.07)		0.54 (0.15-1.99)	
**Sexual encounters in the current week**	**0-9**	102/672(15.2)	1	<0.01	1	<0.01
	**10-20**	54/290(18.6)	1.62 (1.12-2.32)		1.63 (1.13-2.34)	
	**>20**	9/220(4.1)	0.70 (0.36-1.36)		0.72 (0.37-1.38)	
**Age range (years)**	**16 - 25**	96/601(16.0)	1	0.59		
	**26 - 35**	61/469(13.0)	0.82 (0.40-1.71)			
	**>= 36**	8/112(7.1)	0.47 (0.11-2.13)			
**Educational Attainment**	**Primary or less**	104/791(13.1)	1	0.64		
	**Secondary or higher**	61/391(15.6)	1.19 (0.58-2.46)			
**Received material goods or payment in exchange for sex**	**No**	63/353(17.8)	1	0.23		
	**Yes**	78/633(12.3)	0.62 (0.28-1.36)			
	**Missing**	24/196(12.2)				

[1] A total of 165 reports of economic abuse were recorded in 1182 weeks of data collection.

[2] Only variables showing an association with the outcome in univariable analyses were included in the multivariable model.
