# Supplementary material for: Experience of social harms among female sex workers following HIV self-test distribution in Malawi: results of a cohort study
Source: BMC Infect Dis. 2024 Mar 11;22(Suppl 1):978. doi: 10.1186/s12879-024-09178-3 (PMC10926537; doi:10.1186/s12879-024-09178-3)
Supplement: Supplementary file 2 — Additional file 2. Social harms diary [file 12879_2024_9178_MOESM2_ESM.pdf]

BARCODE

(Place Here)

UNITAID·PSI
HIV SELF-TESTING AFRICA

HIV STAR

Social harms monitoring for HIV self-testing in key populations in Malawi

Pictorial Diary

FIELD WORKER ID: □□□/□□□/□□□□

START DATE: □□-□□□-□□□□

Instructions for completing this diary

These instructions will remind you on how to complete this diary.

1. Please complete the daily sheets everyday and the weekly sheet once every week. The first 7 sheets will be completed everyday and the last one sheet will be completed at the end of each week.
2. You will be visited regularly throughout the time you are completing diaries. Feel free to ask any question to the researchers when they visit you. If you experience problems as a result of completing the diary, please call your fieldworker on the phone number below for assistance.

Phone number: **OR**

3. How to complete this diary

A.Please circle an applicable option for each item if you have experienced or not experienced that particular event.

EXAMPLES

*If you had sex today, you will circle 'YES' a box with a check like this:

*If the answer is 'NO' you will circle a box with an 'X'

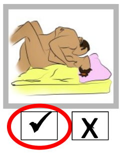

*If you had sex with a client who paid money, you will circle the option with money like this:

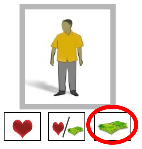

*If you sometimes used a condom with a partner on that day, you will circle an option with a 'half condom' like this:

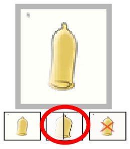

4. PLEASE keep this diary in a secure location.

Thank you for completing this diary.

INTERPRETATION OF DAIRY QUESTIONS

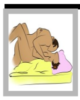

Did you have sex today?

Answer options for this and most questions

1 ✓ YES   2 X NO

Answer ‘YES’ or ‘NO’ to this question everyday.

Note: If the answer is ‘YES’ to this question and you only had sex with one partner that day, COMPLETE the first row and **COMPLETE** ‘NO’ on having sex with a second partner in the second row. If you had sex with 2 partners, COMPLETE the first row. Also indicate ‘YES’ on having sex with a second partner then complete the second row. Then complete ‘No’ on having sex with a third partner in the third row.

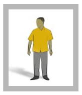

What type of partner did you have sex with today?

Options for this question

1 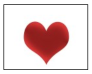 Stable partner

2 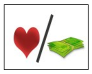 Regular client

3 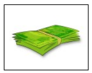 Client

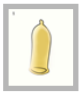

Did you use a condom with this partner today?

Options for this question

Note: Options are about condom use with a partner and not per sexual act.

1 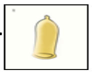 Always used

2 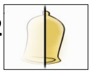 Sometimes used

3 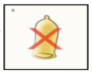 Never used

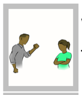

Were you verbally abused by this partner today?

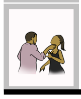

Were you physically abused by this partner today?

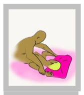

Were you sexually abused by this partner today?

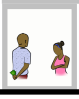

Were you denied economic resources by this partner?

INTERPRETATION OF WEEKLY QUESTIONS

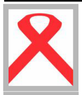

Did you test for HIV this week?

Note: Answer ‘YES’ or ‘NO’ to this question every week. If ‘YES’, please complete applicable rows depending on the type of testing received.

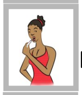

Did you self-test this week?

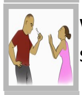

Were you forced to disclose self-test results?

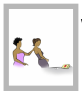

Were you forced to self-test?

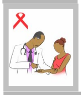

Did you have a blood-based HIV test at a clinic?

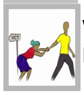

Were you forced to go for an HIV test at a clinic?

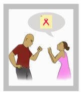

Were you forced to disclose clinic test results?

Diary ID number

DATE: __ __ - __ __ - __ __ __ __ DAY: ______

Note: For each person that you had sex with today, please complete one row.

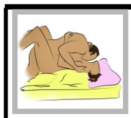

Did you have sex today?

✓ X

2

3

4

5

6

7

8

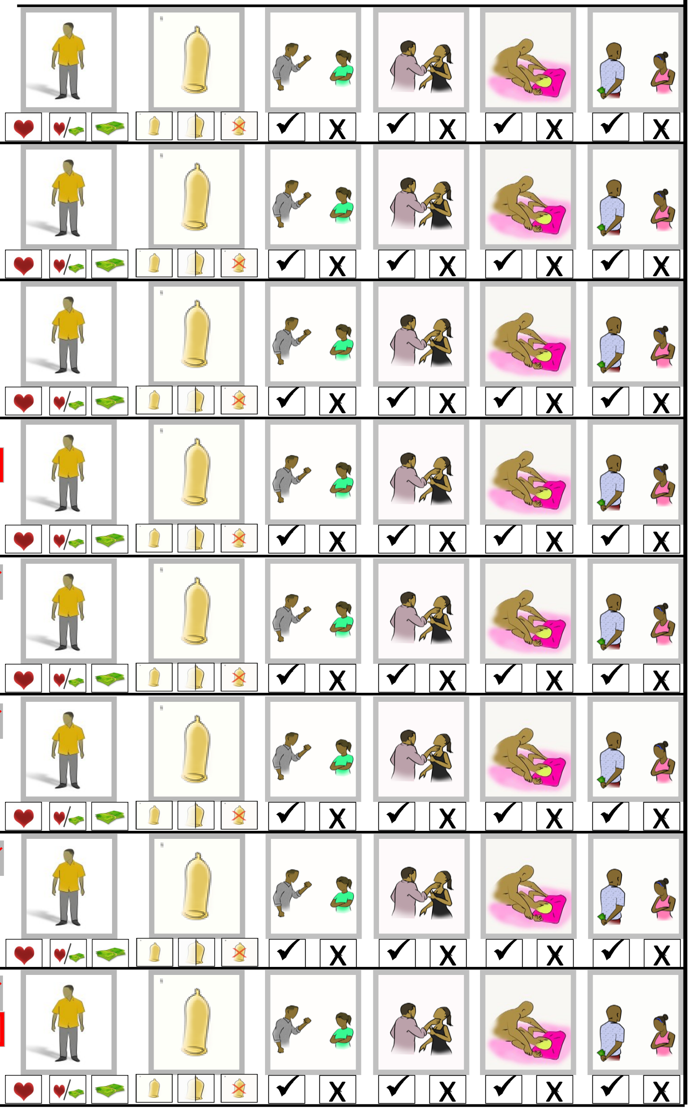

Diary ID number

DATE: __ __ - __ __ - __ __ __ __ DAY:

Note: For each person that you had sex with today, please complete one row.

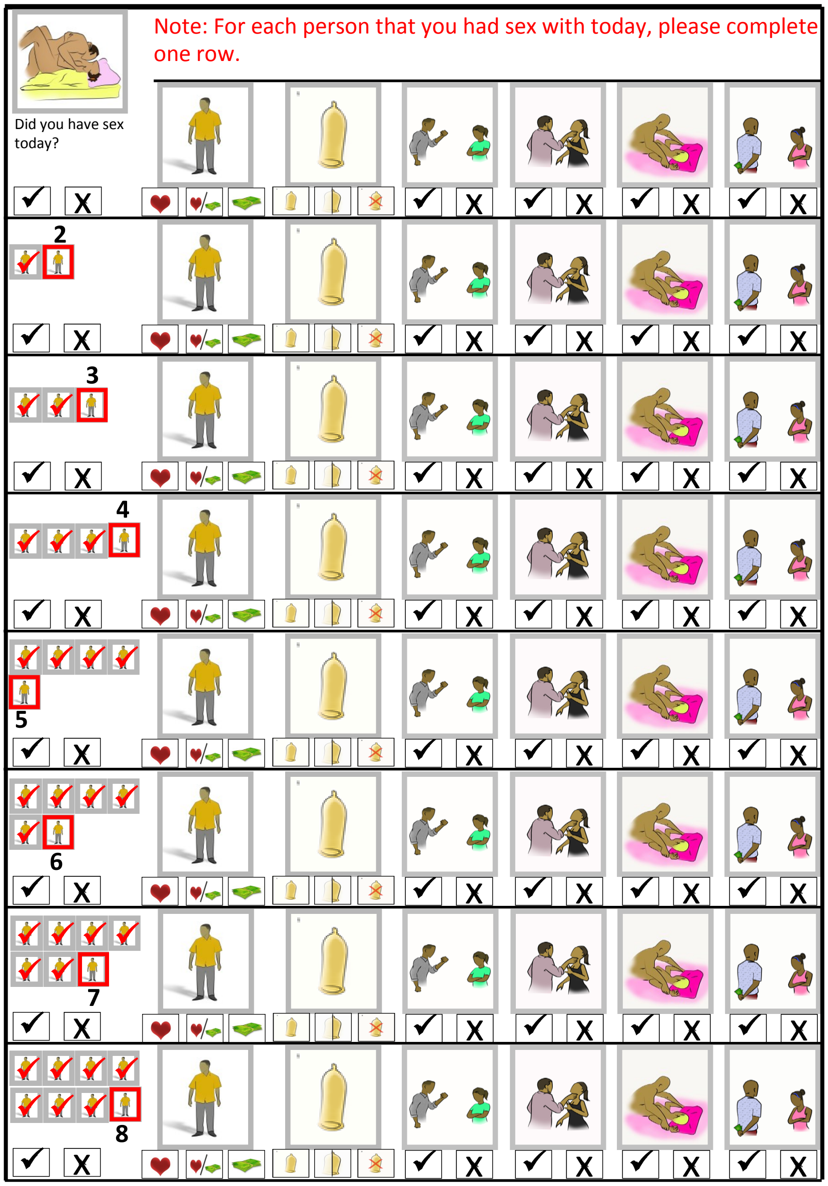

UNITAID-PSI
HIV SELF-TESTING AFRICA

Diary ID number

DATE: ☐☐-☐☐☐-☐☐☐☐ DAY: ☐

Note: For each person that you had sex with today, please complete one row.

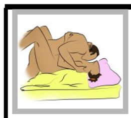

Did you have sex today?

✓ X

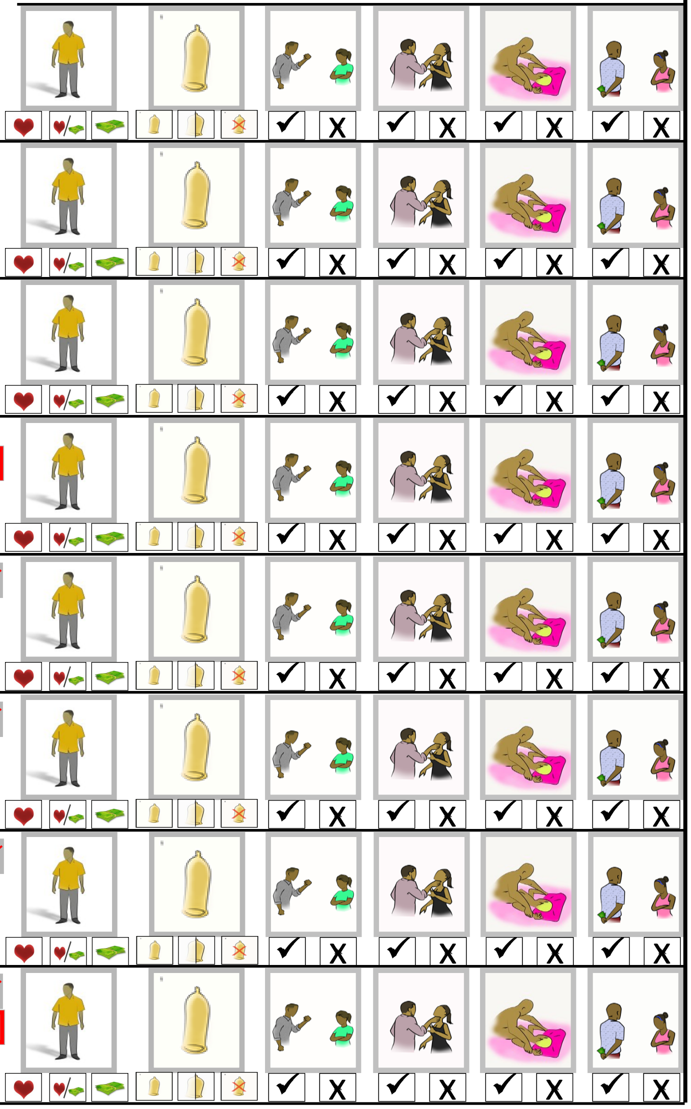

2 ✓ X

3 ✓ X

4 ✓ X

5 ✓ X

6 ✓ X

7 ✓ X

8 ✓ X

UNITAID·PSI
HIV SELF-TESTING AFRICA

DATE: ☐☐-☐☐☐-☐☐☐☐ DAY: ☐

Diary ID number

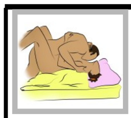

Note: For each person that you had sex with today, please complete one row.

Did you have sex today?

✓ X

2

3

4

5

6

7

8

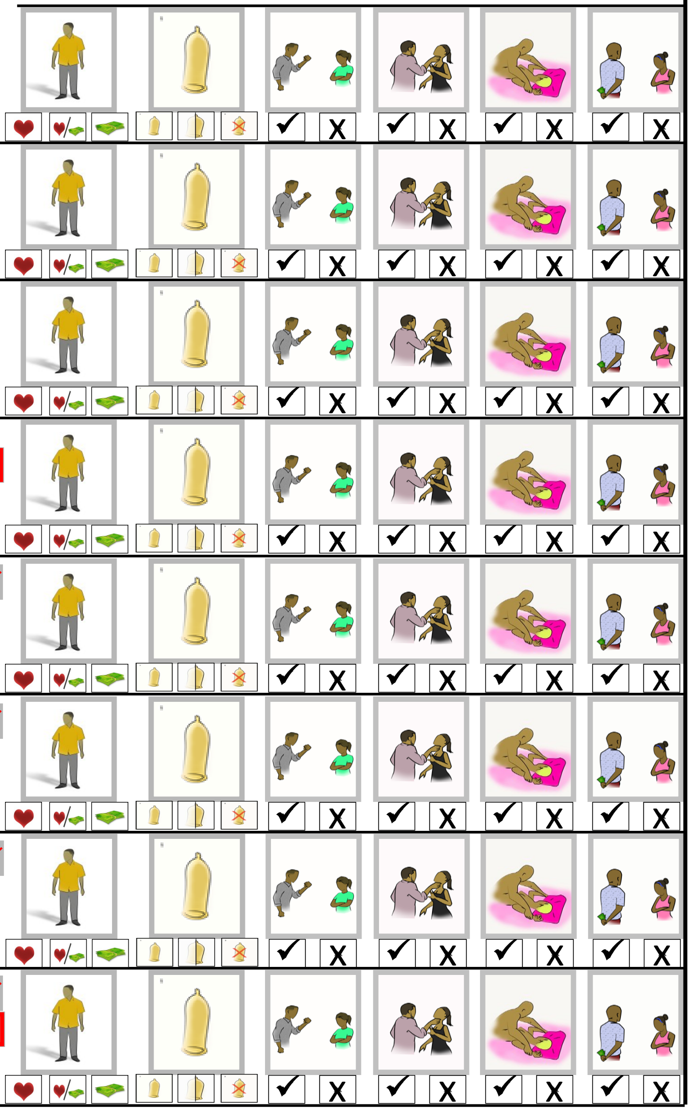

UNITAID-PSI
HIV SELF-TESTING AFRICA

Diary ID number

DATE: __ __ - __ __ - __ __ __ __ DAY: ________

Note: For each person that you had sex with today, please complete one row.

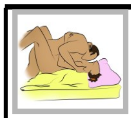

Did you have sex today?

✓ X

2

3

4

5

6

7

8

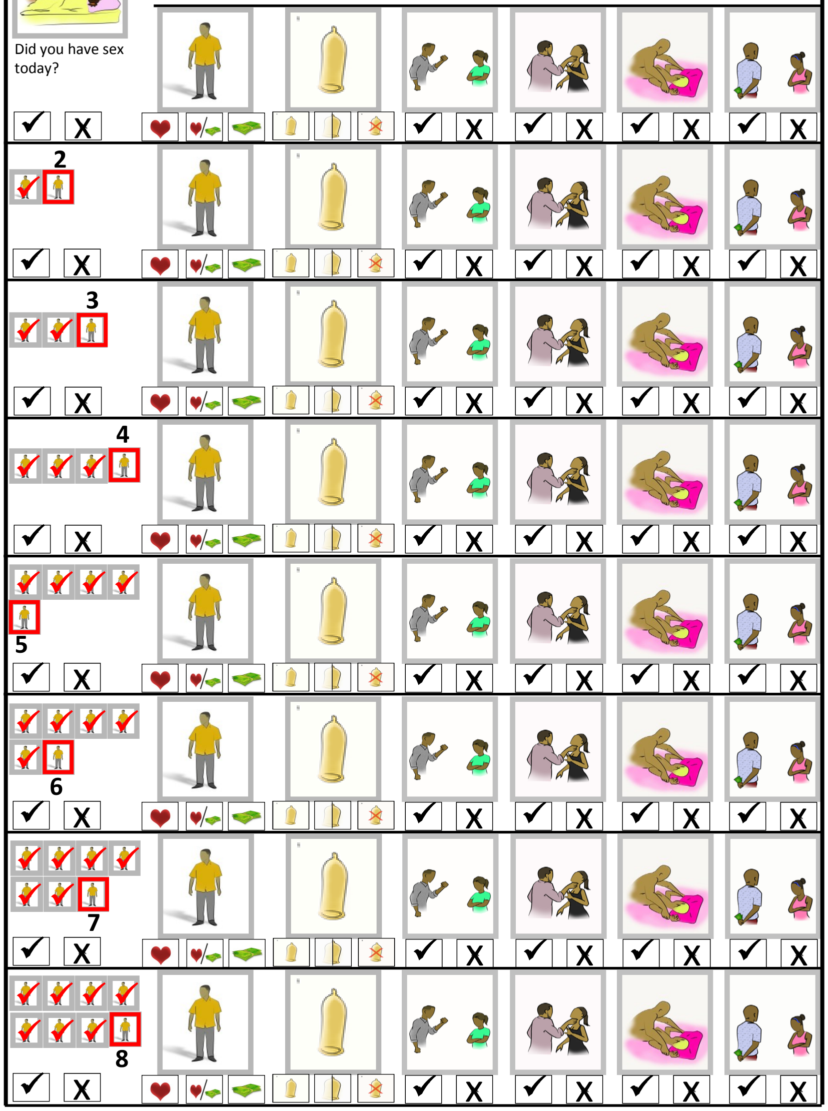

UNITAID-PSI
HIV SELF-TESTING AFRICA

Diary ID number

DATE: __ __ - __ __ - __ __ __ __ DAY: ____________

Note: For each person that you had sex with today, please complete one row.

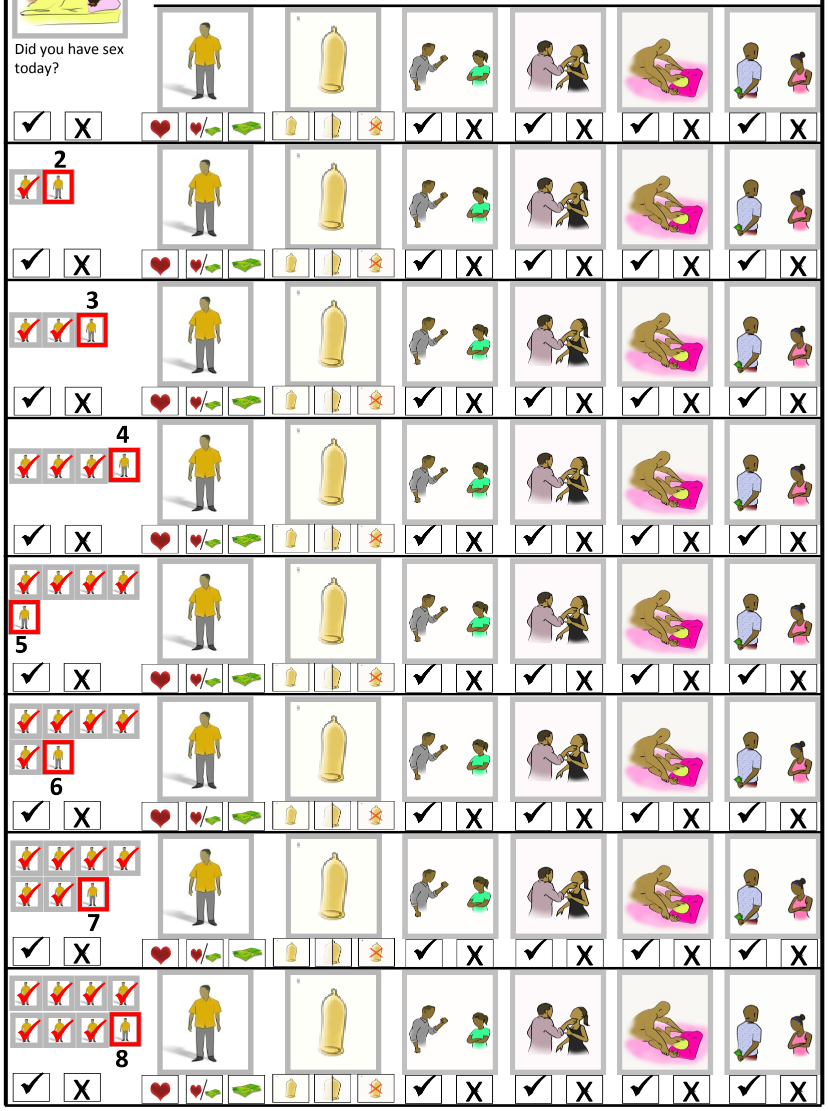

Diary ID number

DATE: __ __ - __ __ __ - __ __ __ __ DAY:

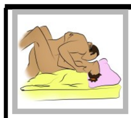

Did you have sex today?

Note: For each person that you had sex with today, please complete one row.

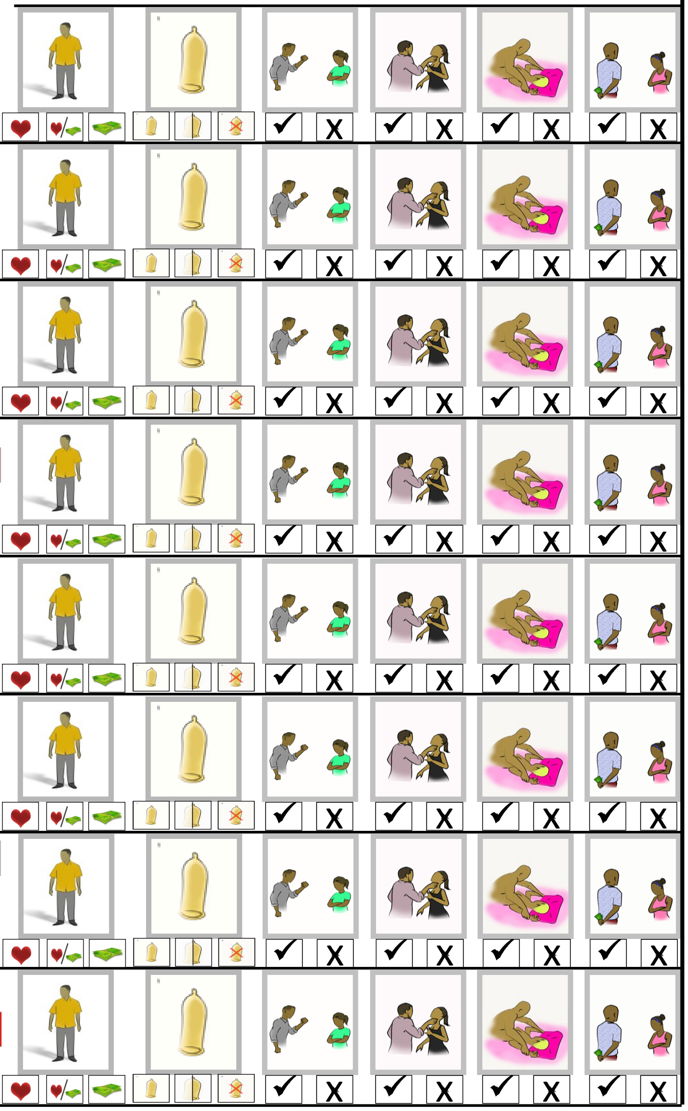

2

3

4

5

6

7

8

UNITAID·PSI HIV SELF-TESTING AFRICA

DATE: _ _ - _ _ _ - _ _ _ _ DAY: ______

Diary ID number

Note: Please ANSWER the question about HIV testing EVERY WEEK. If 'YES' to this question, COMPLETE row '1' if you 'self-tested ' this week OR row '2' if you tested for HIV at a facility OR row '1' and row '2' if you had both.

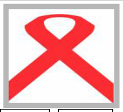

✓ ✗

Did you test for HIV this week?

1

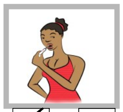

✓ X

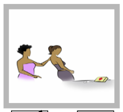

✓ X

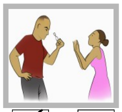

✓ X

2

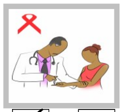

✓ X

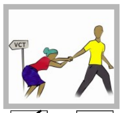

✓ X

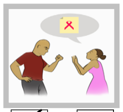

✓ X

UNITAID-PSI
HIV SELF-TESTING AFRICA

DATE: __ __ - __ __ - __ __ __ __   DAY: ________

Diary ID number

Note: For each person that you had sex with today, please complete one row.

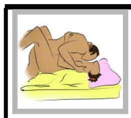

Did you have sex today?

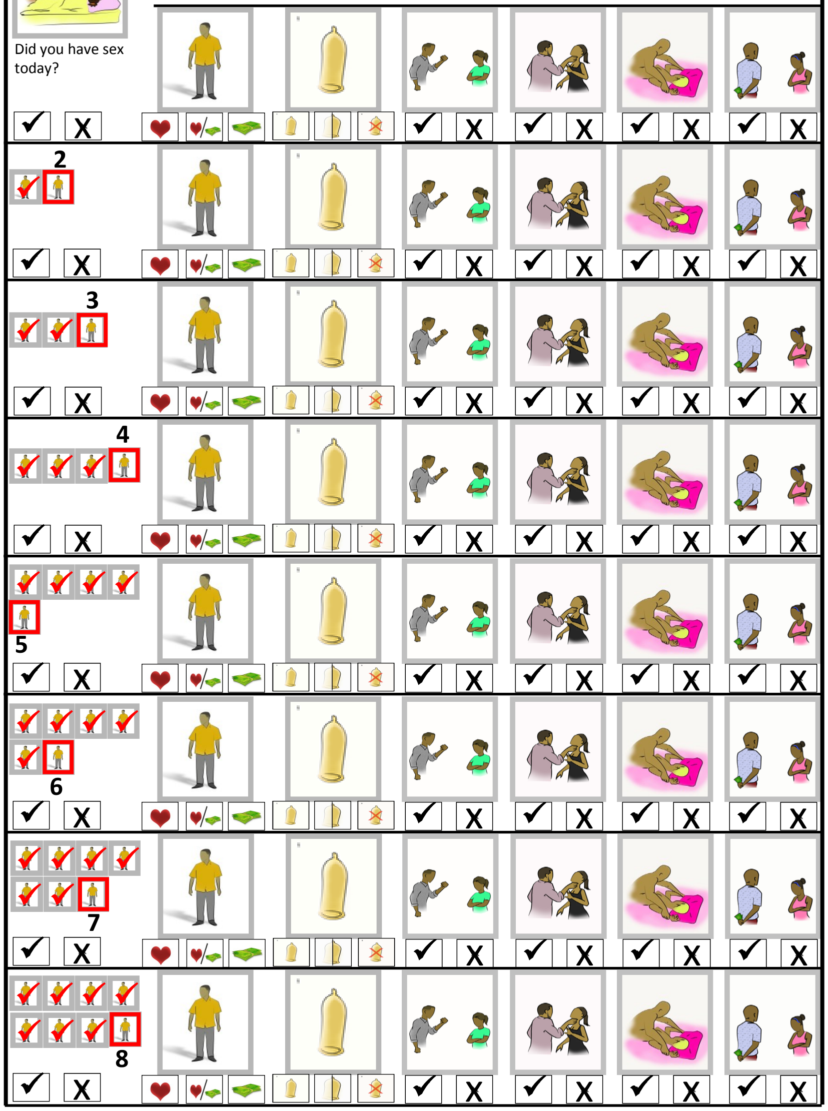

UNITAID·PSI
HIV SELF-TESTING AFRICA

Diary ID number

DATE: ☐☐-☐☐-☐☐☐☐ DAY: ______

Note: For each person that you had sex with today, please complete one row.

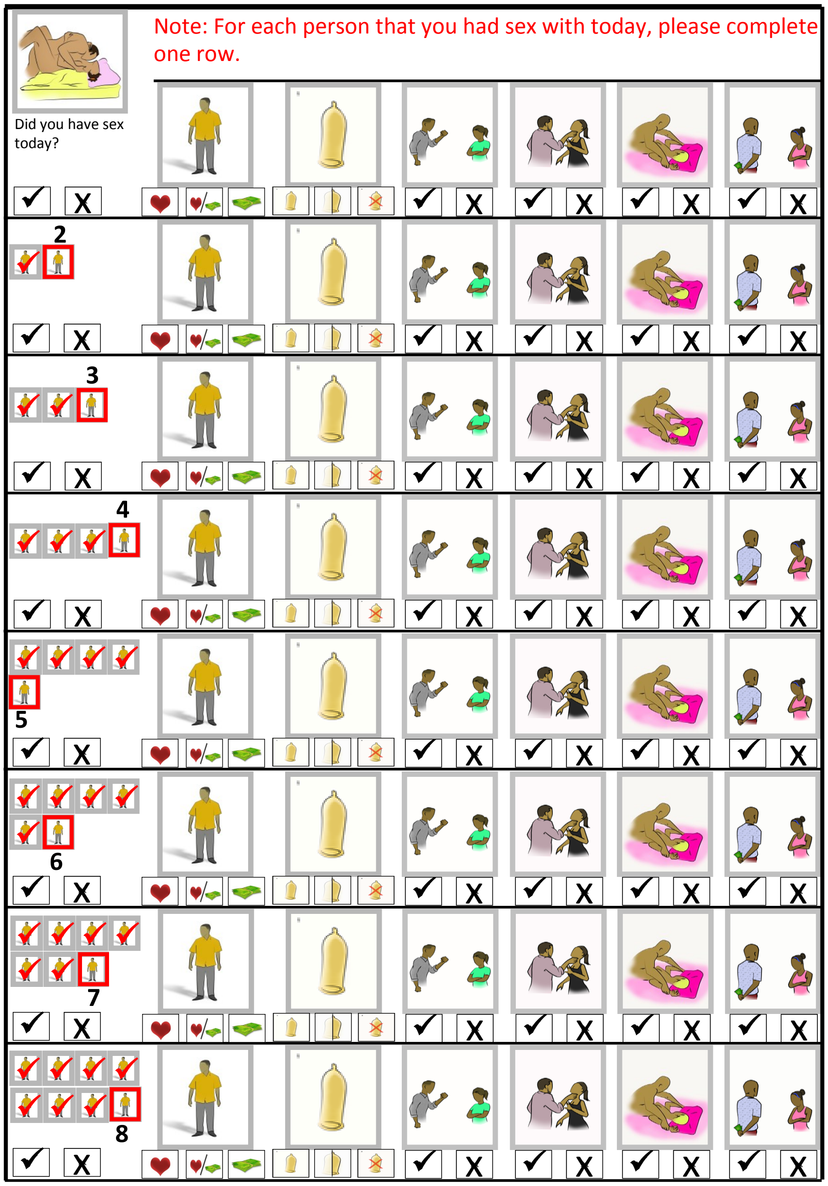

UNITAID·PSI HIV SELF-TESTING AFRICA

DATE: _ _ - _ _ _ - _ _ _ _ DAY:

Diary ID number

Note: For each person that you had sex with today, please complete one row.

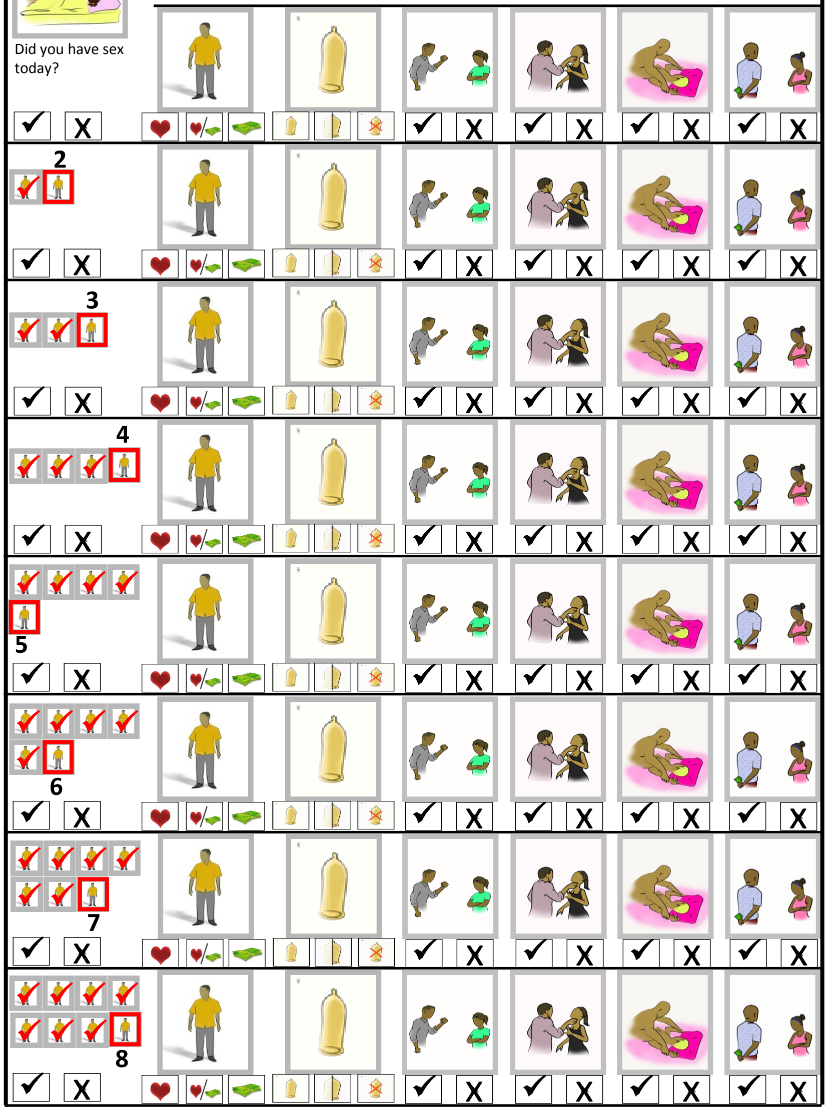

UNITAID-PSI
HIV SELF-TESTING AFRICA

Diary ID number

DATE: __ __ - __ __ - __ __ __ __ DAY:

Note: For each person that you had sex with today, please complete one row.

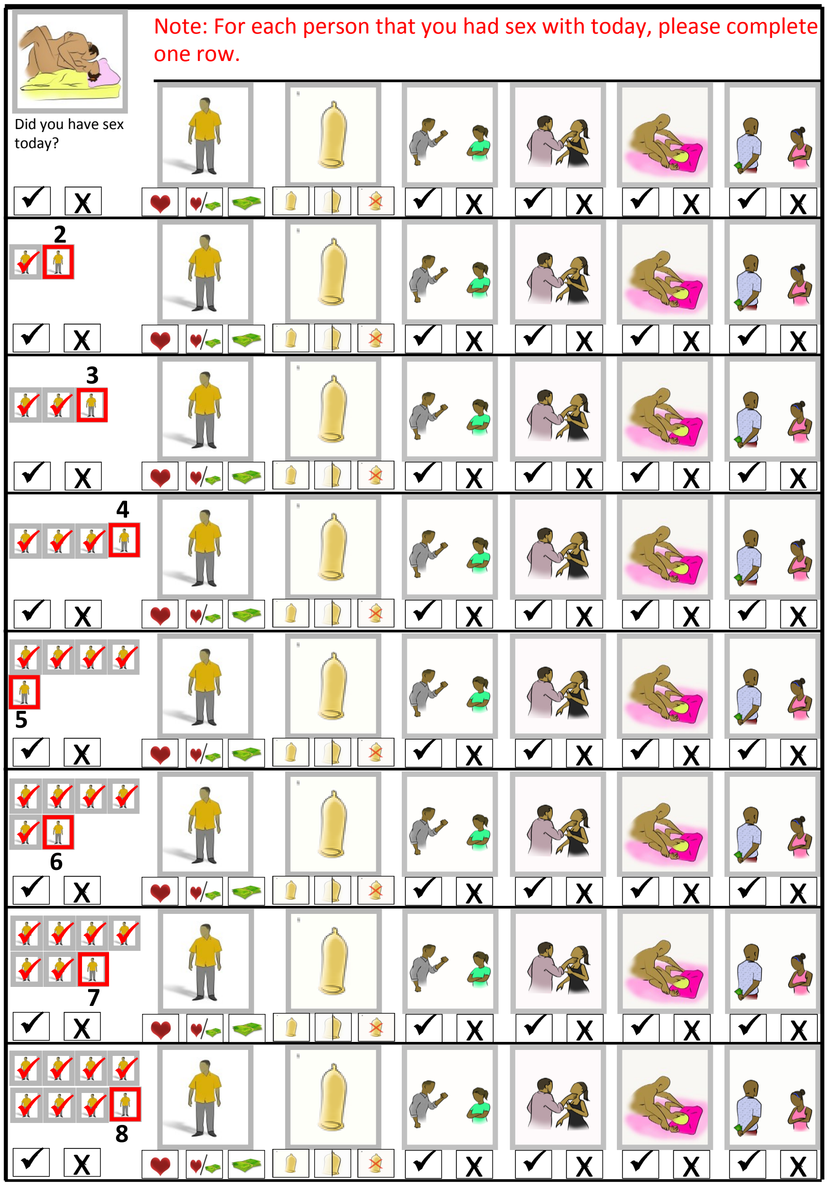

UNITAID-PSI
HIV SELF-TESTING AFRICA

DATE: _ _ - _ _ - _ _ _ _ DAY:

Diary ID number

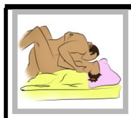

Note: For each person that you had sex with today, please complete one row.

Did you have sex today?

✓ X

2

✓ X

3

✓ X

4

✓ X

5

✓ X

6

✓ X

7

✓ X

8

✓ X

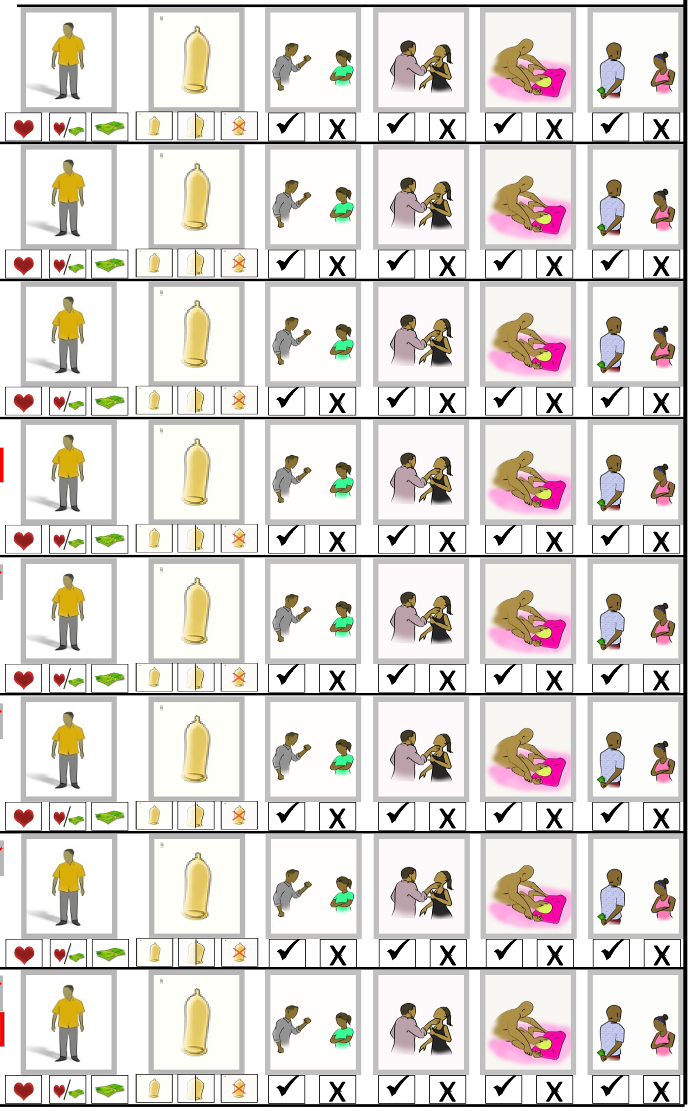

Diary ID number

DATE: [ ][ ]-[ ][ ][ ]-[ ][ ][ ][ ] DAY: [ ]

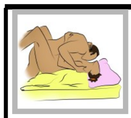

Note: For each person that you had sex with today, please complete one row.

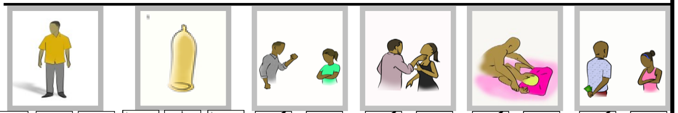

Did you have sex today?

2

3

4

5

6

7

8

UNITAID·PSI
HIV SELF-TESTING AFRICA

DATE: __ __ - __ __ - __ __ __ __ DAY: ____________

Diary ID number

Note: For each person that you had sex with today, please complete one row.

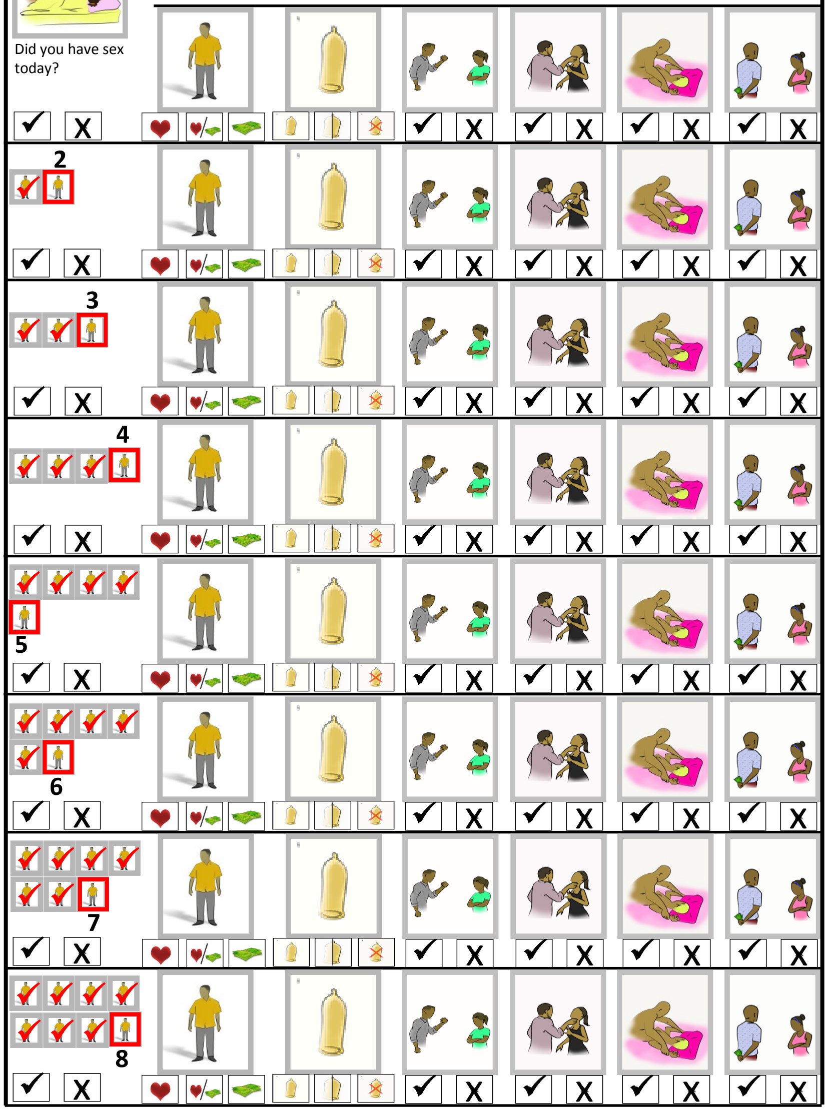

Diary ID number

UNITAID·PSI HIV SELF-TESTING AFRICA

DATE: __ __ - __ __ - __ __ __ __ DAY: ________

Note: Please ANSWER the question about HIV testing EVERY WEEK. If 'YES' to this question, COMPLETE row '1' if you 'self-tested ' this week OR row '2' if you tested for HIV at a facility OR row '1' and row '2' if you had both.

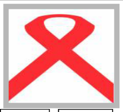

✓ ✗

Did you test for HIV this week?

1

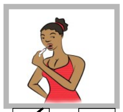

✓ X

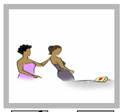

✓ X

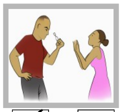

✓ X

2

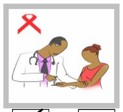

✓ X

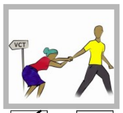

✓ X

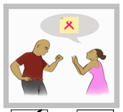

✓ X
