# Supplementary material for: Experience of social harms among female sex workers following HIV self-test distribution in Malawi: results of a cohort study
Source: BMC Infect Dis. 2024 Mar 11;22(Suppl 1):978. doi: 10.1186/s12879-024-09178-3 (PMC10926537; doi:10.1186/s12879-024-09178-3)
Supplement: Supplementary file 3 — Additional file 3. ACASI questionairre [file 12879_2024_9178_MOESM3_ESM.pdf]

PS904 - ACASI
STAR Malawi Version 2.0 - 02/05/16

A. Survey

To be completed by interviewer

Question No.	Construct	Variable name	Wording of question (English)	Wording of question (Chichewa)	Data type	Data type (Chichewa)	Skip	Range
AC01	Clinic ID	clinicid	Village ID		Choose from list of clinics			
AC02	Village ID	villageid	Interview ID		Choose from list of villages			
AC03	Household ID/Barcode	hhbarcode	Household ID		Randomly generated unique ID			
AC04	Interviewer ID	interviewerid	Individual ID		Choose from list of interviewers, or auto set by signing into tablet			
AC05	Date of interview	intdate	Interview date		Current date			Should be set to date in device
AC06	Start time	starttime	Start time		Time			Should be set to time in device
AC07	Lat-Long	latlong	Lat-Long		Automatic			

PS904 - ACASI
STAR Malawi Version 2.0 - 02/05/16

B. Sociodemographic

To be completed by all individuals
Prompt: Tsopano ndikufunsani mafunso okhudzana ndi inuyo.
Prompt: I would like to ask you a few questions about you.

Question No.	Construct	Variable name	Wording of question (English)	Wording of question (Chichewa)	Data type	Data type (Chichewa)	Skip	Range
AC08	Individual ID	individ	Unique identifier/barcode		Randomly generated unique ID			
AC09	Date of birth	respdob	What is your date of birth?	Tsiku lanu lakubadwa	Date		If non-missing, skip to edu	1/1/1926 - 1/1/2000 (or previously used range)
AC10	Age in years	respageyrs	How old are you?	Muli ndi zaka zingati?	Number			16-99
AC11	Educational attainment	edu	What was the highest level of education that you have completed?	Maphunziro anu munafika nawo patali bwanji?	1 No formal schooling<br>2 Primary incomplete or complete<br>3 Some secondary education<br>4 Secondary school complete<br>5 College or higher	1 Sindinapite ku sukulu iliyonse<br>2 Sindanalize kapena kumalisa pulayimale<br>3 Sindinamalize sekondale<br>4 Ndinamaliza sekondale<br>5 Sukulu ya ukachenjede kapena kuposera		
AC12	Literacy	literate	Can you read a newspaper or letter?	Kodi mumatha kuwerenga nyuzi pepala kapena kalata?	1 Yes<br>2 No	1 Eya<br>2 Ayi		
AC13	Employment status	salary	Do you receive a regular salary? By regular salary, I mean money that is paid by the employer daily, weekly, or monthly during the last two months or longer?	Kodi mumalandira malipilo kuchoka kwa okulembani ntchito? Malipilo ake apa tsiku, pa sabata kapena apa mwezi mu miyezi iwiri yapitayi kapena kupyola?	1 Yes<br>2 No	1 Eya<br>2 Ayi		
AC14	Self-reported health	srhealth	How do you rate your general health?	Kodi nthanzi lanu mumaliwona bwanji	1 Very good<br>2 Good<br>3 Fair<br>4 Poor	1 Lili bwino kwambiri<br>2 Lili bwino<br>3 Lili bwino pang'ono<br>4 Silili bwino		
AC15	Marital status	marital	What is your current marital status?	Kodi pankhani yokhudza banja, mwa izi ndi chiti chimene chikulongosola bwino za inuyo?	1 Married or living as married<br>2 Never married<br>3 Widowed/separated/divorced	1 Wokwatira kapena Kukwatiwa<br>2 Sanakwatiwe kapena kukwatirapo<br>3 wofedwa/tinasiyana	If marital=2, skip to children	
AC16	Partnership	partnerlength	How long have you been together with your spouse or partner [or first spouse/ partner for persons with multiple spouses]?	Kodi mwakhala pa banja ndi mwamuna/mkazi/ wanu/chibwezi chanu kwa nthawi yayitali bwanji? (Kapena mwamuna/mkazi wanu woyamba kwa omwe ali ndi mkazi/mwamuna oposera awiri)	1 <1 year<br>2 1-5 years<br>3 More than 5 years	1 Kochepera chaka chimodzi<br>2 Pakati pa chaka chimodzi kufika zaka Zisanu<br>3 Kupyola zaka Zisanu		
AC17	Living with spouse/partner	livepartner	Are you currently living with your spouse/ partner?	Kodi panthawi ino mukukhala ndi mkazi/mwamuna wanu/chibwezi chanu?	1 Yes<br>2 No	1 Eya<br>2 Ayi		

PS904 - ACASI
STAR Malawi Version 2.0 - 02/05/16

L. Sexual behavior - extended

To be completed by all consenting to participate within the household

Prompt: Now I would like to ask you questions about your sexual activity in order to gain a better understanding of some important life issues. Let me asure you that your answers are completely confidential and will not be told to anyone. If we should come to any question that you don't want to

Question No.	Construct	Variable name	Wording of question (English)	Wording of question (Chichewa)	Data type	Data type (Chichewa)	Skip	Range
AC18	In partnership YN	steadyyn	Do you have a steady partner?	Muli ndi chibwenzi chokhazikika?	1 Yes<br>2 No	1 Eya<br>2 Ayi	If no, skip to otheryn	
AC19	Steady partner - count	steadyct	If yes, how many steady partners have you had sex with in the last 3 months?	Ngati zili choncho mwagonana ndi zibwezi zanu zokhazikika zingati miyezi itatu yapitayi?	Number			1-25
AC20	Condomless sex indicator for each steady partner	partnocond_X	In the past 3 months, how often have you not used condoms with this steady partner?	Pa miyezi itatu imeneyi mwagwilitsa ntchito condom ndi abwenzi anu okhazikika kangati?	1 Condoms every time<br>2 Condoms some of the time<br>3 Condoms never used<br>99 Don't want to answer	1. Pathawi iliyonse<br>2. Nthawizina timagwiritsira ntchito ma kondomu nthawi zina ayi<br>3. sitigwiritsira ntchito ma kondomu<br>99. sindikufuna kuyankha	Note that this question will have to be asked for each partner from AC19	
AC21	Non-steady partner YN	otheryn	[Apart from your steady partner(s)], have you had sex with anyone else in the last 3 months? (Modify wording as needed based on response to steadyyn)	Kupatula abwenzi/zibwenzi zanu zokhazikika mwagonanapo ndi wina aliyense pa miyezi itatu yapitayi?	1 Yes<br>2 No	1 Eya<br>2 Ayi	If no, skip to next section	
AC22	Count of non-steady partners with condomless sex	othernocond	If yes, with how many people apart from your steady partner have you had sex **without using a condom**, even if was only on one occasion?	Ngati zili choncho ndi anthu angati kupatula chibwenzi chanu chokhazikika amene mwagonana nawo opanda chitetezo olo uli ulendo umodzi?	Number	Number		1-25
AC23	Sex work	sexwork	Have you received money, food, material things or bills paid for you in exchange for sex, in the last 3 months?	Munayamba mwalandirapo ndalama, chakudya, zinthu kapena kulipililidwa ma bilu chifukwa chogonana ndi mwamuna pa miyezi itatu yapitayi?	1 Yes<br>2 No	1 Eya<br>2 Ayi	If no, skip to next section	
AC24	Sex work- count	sexworkct	If yes, how many partners have you had sex with in exchange for money, food, material things or bills paid for you?	Ngati zili choncho, ndi amuna angati amene anakupatsani ndalama, chakudya, zinthu kapena kukulipilirani ma bilu chifukwa chogonana nawo?	Number	Number		
AC25	condom use for each sex work partner	swconduse	The last time you received money, food, material things or bills paid for you in exchange for sex, was a condom used?	Nthawi yomaliza imene munalandira ndalama, chakudya, zinthu kapena kulipililidwa ma bilu chifukwa chogonana ndi mwamuna munagwiritsa ntchito kondomu?	1 Yes<br>2 No	1 Eya<br>2 Ayi		

PS904 - ACASI
STAR Malawi Version 2.0 - 02/05/16

C. HIV testing

To be completed by all individuals
Prompt: Tsopano ndikufunsani mafunso ena okhudzana ndi kuyezesa kwanu kwa HIV
Prompt: Now I would like to ask you some questions about your experiences testing with HIV.

Question No.	Construct	Variable name	Wording of question (English)	Wording of question (Chichewa)	Data type	Data type (Chichewa)	Skip	Range
AC23	Ever tested for HIV	evertest	Have you ever been tested for HIV?	Munayamba mwayezesapo HIV?	1 Yes<br>2 No	1 Eya<br>2 Ayi	If yes skip to yrtestcount	
AC24	Why not tested?	whynottest_X	What best describes why you haven' t tested for HIV?	Ndichifukwa chiti choposa zina zonse, chomwe munganene kuti, ndichomwe inu simunayezetsepo HIV?	(Select all that apply)<br>A Not at risk of being HIV positive<br>B Fear of testing positive<br>C Fear of stigma and discrimination from testing for HIV<br>D Partner won't let me test<br>E Parents won't let me test<br>F Other family won't let me test<br>G Do not have money to test<br>H Cannot take time off work to test<br>I Do not have time to test<br>J Not a dignified thing to do at my age<br>K Poor quality of HTC services, including lack of confidentiality<br>L Other reason	A Kusakhala pachiopsezo chokhala ndi HIV<br>B Mantha opezeka ndikachilombo koyambisa HIV<br>C Mantha osalidwa, komanso zuzisaritsa ndekha, kamba koyezesa.<br>D chibwenzi changa sichimandiloleza kupita kokayezetsa<br>E Makolo samandiloleza kupita kokayezesa<br>F Abale ena sangandiloleze kuti ndikayezese<br>G Ndilibe ndalama zokayezetsera<br>H Sindingapeze mpata wokayezesa chifukwa cha ntchito.<br>I Ndilibe nthawi yopita kukayezetsa<br>J Sichinthu choyenera kuchita pamsinku wanga/zaka zanga<br>K Anthu opereka uphungu wa HIV samasunga chinsinsi, komanso kaperekedwe kachithandizo cha HTC sikabwino.<br>L Chifukwa china chilichonse		
AC25	Testing in last twelve months	yrtestcount	In the last 12 months, how many times have you tested for HIV?	Pamiyezi 12 yadutsayi, mwayezetsapo kangati?	Number			0-15
AC26	Lifetime test count	lifetestcount	In total, how many HIV tests have you had in your lifetime	Mophatikiza, munayezetsapo kangati mmoyo mwanu wonse?	Number			1-50 - value must be greater than yrtestcount

PS904 - ACASI
STAR Malawi Version 2.0 - 02/05/16

D. HIV self-test

To be completed by all individuals
Prompt: Tsopano ndikufunsani mafunso ena okhudzana ndi kuziyeza nokha HIV
Prompt: Now I would like to ask you some questions about your experiences testing with HIV self-testing

Question No.	Construct	Variable name	Wording of question (English)	Wording of question (Chichewa)	Data type	Data type (Chichewa)	Skip	Range
AC27	Self test within past 12 months	selftest12mos	Within the past 3 months, have you used a self-test to test for HIV?	Pa miyezi 3 yadutsayi , mwagwiritsirako nchiti chipangizo choziyezera nokha, poziyeza HIV	1 Yes<br>2 No	1 Eya<br>2 Ayi		
AC28	Initiation of test	testinit_X	Who initiated the self-test?	Ndindani anayambitsa/anabweretsa ganizo loyezesa?	1 Yourself<br>2 Your partner<br>3 Peer distributor<br>4 Other	1 Inuyo<br>2 Wachikondi wanu<br>3 Anzanu ogawa zipangizo zoyezera<br>8 Zina		
AC29	Discussion with partner	discusspart	Did you discuss testing with your partner before you had the self-test?	Kodi munakambirana zokhuzana ndi kuyezesa ndi wachibwezi chanu musanayezese?	1 Yes<br>2 No	1 Eya<br>2 Ayi		
AC30	Self-test - anyone present?	selftestalone	Was anyone else with you when you self-tested?	Kodi panali munthu wina aliyense pamene mumaziyeza nokha?	1 Yes<br>2 No	1 Eya<br>2 Ayi	If no, skip to testinit_X	
AC31	Self-test - who present?	selftestpresent	Who was present when you self-tested?	Ndindani analipo nthawi imene mumaziyeza nokha?	(Check all that apply)<br>A Spouse or partner<br>B Other family member<br>C Friend<br>D Employer<br>E Peer distributor<br>F Health care worker<br>G Other	A Wachikondi<br>B M'bale wabanja limodzi<br>C Mzanu<br>D Bwana/wolemba ntchito<br>E Wopereka zipangizo zoziyezera<br>F Anzanu ogawa zipangizo zoyezera<br>G Wina	If not A, skip to testinit_X	
AC32	Self-test with partner	selftestpartner	Did you self-test at the same time as your partner also self-tested?	Kodi munaziyeza nthawi imodzi imene chibwezi chanu chimaziyeza?	1 Yes<br>2 No	1 Eya<br>2 Ayi	Skip to testinit_X if selftest_X=yes (as we already have partner info)	
AC33	Results of last HIV test	knowres	You don't have to tell me if you don't want to, but what were the results of your self-test?	Simukuyenera kunduwuza ngati simukufuna, koma zosatira za kuyezesa kwanu zinali zotani?	1 Positive<br>2 Negative<br>3 Indeterminate<br>4 Prefer not to reveal	1 Mulinako kachirombo<br>2 Mulibe kachirombo<br>3 Zosaziwika bwinobwino<br>4 Simukufuna kuwulura		
AC34	Regret testing immediately after testing	regretimm	Aside from the results, did you have any regrets about your self-test immediately after you completed the test?	Kupatula zosatira zoyezesa, kodi munali ndi Kunong'oneza bondo kwina kulikonse mutangoyezesa kumene/mutangomaliza kumene kuyezesa?	1 Yes<br>2 No	1 Eya<br>2 Ayi		
AC35	Regret testing now	regretnow	Looking back on your self-test now, do you regret taking this test now?	Mukayang'ana kuyezesa kwanu kwapanop, kodi mukunong'oneza bondo panopo chifukwa chakuyezesaku?	1 Yes<br>2 No	1 Eya<br>2 Ayi		
AC36	Problems caused by test	relprob	Were there any problems in your relationship caused by your self-test?	Kodi panali mavuto ena alionse pa ubwenzi wanu kamba kuziyeza nokha?	1 Yes<br>2 No	1 Eya<br>2 Ayi		
AC37	Forced to test	testforce	If you were forced to self-test, who forced you?	Ngati munakakamizidwa kuti muyezese, anakukakamizani ndani?	1 Not forced to test<br>2 Partner/spouse<br>3 Parent<br>4 Other family member<br>5 Employer<br>6 Other<br>7 Prefer not to say	1 Simunakakamizidwe<br>2 Chibwezi/amuna anga<br>3 Kholo<br>4 M'bale wabanja limodzi<br>5 Bwana/wolemba ntchito<br>6 Anthu ena<br>7 Simukufuna kunena kanthu		
AC38	Forced disclosure	forcedisc	If you were forced to disclose your HIV test results to another person, who forced you?	Ngati munakakamizidwa kuwulura zotsatira za kuyezesa kwanu kwa munthu wina, kodi anakukakamizani ndi ndani?	1 Not forced to disclose<br>2 Partner/spouse<br>3 Parent<br>4 Other family member<br>5 Employer<br>6 Other	1 Sindinakakamizidwe<br>2 Wachikondi<br>3 Kholo<br>4 M'bale wabanja limodzi<br>5 Bwana/Wolemba ntchito<br>6 Anthu ena		
AC39	Actions after testing	aftertest	What actions did you take after the self-test?	Kodi munapanga chani mutamaliza kuziyeza nokha?	(Check all that apply)<br>A Confirmed result<br>B Went for HIV care<br>C Got condoms<br>D Did not do anything<br>E Don't want to answer	A Kutsimikiza zosatira<br>B Kupita kukapeza chithandizo cha HIV<br>C Kupeza makondomu<br>D Simunapange china chilichonse<br>E Simukufuna kuyankha		
AC40	Partner status known	partnerstatknwn	Do you know the result of your partner's most recent HIV test?	Kodi mukudziwa zotsatira zakuyezesa kumene kwachitika posachedwa kwakachirombo ka HIV za chibwezi chanu?	1 Yes<br>2 No	1 Eya<br>2 Ayi		
AC41	Partner knows respondent's status	ownstatknwn	Does your partner know your HIV status?	Kodi chibwezi chanu chikudziwa m'mene mthupi mwanu muliri pankhani yokhudza kachirombo ka HIV?	1 Yes<br>2 No	1 Eya<br>2 Ayi		
AC42	Prefered mode of testing	prefmode	Which would you want most to be your next test?	Kodi ndinjira iti imene mungayikonde kwambiri mukamazayezesanso patsogolo pano?	1 Hospital, clinic or health center<br>2 VCT centre<br>3 Mobile HTC<br>4 At-home HTC<br>5 Self-testing with counsellor present<br>6 Self-testing without counsellor present	1 Chipatala<br>2 Malo a VCT<br>3 Kuyezesa kichitikira mudera/m'mudzi<br>4 Kunyumba<br>5 Kuziyeza nokha pali phungu<br>6 Kuziyeza nokha opanda phungu		
AC43	Recommend self test	recselftest	Would you recommend self-testing to a friend or family member?	Mungamulangize mzanu kapena wachibale, kuti agwiritsire ntchito njira yoziyeza wekha?	1 Yes<br>2 No	1 Eya<br>2 Ayi		

PS904 - ACASI
STAR Malawi Version 2.0 - 02/05/16

D. HIV self-test

To be completed by all individuals
Prompt: Tsopano ndikufunsani mafunso okhudzana ndi zinthu zomwe munakumana nazo mutayezesa HIV
Prompt: Now I would like to ask you some questions about your experiences testing with HIV.

Question No.	Construct	Variable name	Wording of question (English)	Wording of question (Chichewa)	Data type	Data type (Chichewa)	Skip	Range
AC44	ART current use	artcurruse	Are you currently using ART or ARV drugs?	Kodi panthawi ino mukumwa mankhwala otalikitsa moyo kapena ma ARV?	1 Yes<br>2 No	1 Eya<br>2 Ayi	If yes, skip to artclinic	
AC45	ART previous use	artprevuse	Have you ever previously taken ART or ARV drugs?	Kodi m'mbuyomu munayamba mwamwapo mankhwala otalikitsa moyo kapena ma ARV?	1 Yes<br>2 No	1 Eya<br>2 Ayi	If artcurruse = 2 and artprevuse = 1, go to artwhydisconti n	
AC46	ART - why discontinued	artwhydisconti n	What is the main reason why you have stopped taking ART OR ARVs?	Ndi chifukwa chani cheni cheni chimene munasiya kumwa mankhwala otalikitsa moyo kapena ma ARV?	1 I thought I was experiencing side effects of the drugs so I decided to stop<br>2 I stopped attending the clinic to get the drugs because I couldn't afford to attend<br>3 I stopped attending the clinic to get the drugs because I could not afford to pay for transport to attend<br>4 Other reason	1 Ndimaonangati sindikuyanjana ndimakhwala nde ndinasiya.<br>2 Ndinasiya kupita kuchipatala kukatenga mankhwala chifukwa chakuti sindimatha kukwanisa kupitako<br>3 Ndinasiya kupita kuchipatala kukatenga mankhwala chifukwa chakuti ndinalibe ndalama zopitira kuchipatala<br>4 Cholinga china		

PS904 - ACASI
STAR Malawi Version 2.0 - 02/05/16

E. IPV

Prompt: Mafunso awa, ndiokhudzana ndi zinthu zosiyanasiyana zimene azimayi komanso azibambo amakumana nazo, zoti chibwenzi chawo cha panopa kapena chakale chinawachitapo.
Prompt: The next questions are about things that happen to many women and men and that your current partner or any other partner may have done to you.

Question No.	Construct	Variable name	Wording of question (English)	Wording of question (Chichewa)	Data type	Data type (Chichewa)	Skip	Range
AC47	Psychological IPV - YN	ipvpsychyn	In the past 3 months did your partner do the following to you? Insulted you; made you feel bad; belittled, humiliated, scared you (yelled or smashed things), or threatened to hurt you?	Pamiyezi 3 yadusayi, chibwenzi chanu chapangako zinthu izi: kukuyalusani, kukupangisani kuti mumve ngati ndinu olakwa kapena oipa, kukuchepetsani, kukuopsyezani (kukukalipirani/kuphwanya zinthu), kuopsyeza kuti akupwetekani munjira iliyonse.	1 Yes<br>2 No<br>99 Decline to answer	1 Eya<br>2 Ayi<br>99 Akana kuyankha	If no, skip to ipvphysicyn	
AC48	Psychological IPV - number of times	ipvpsychct	In the past month, would you say this has happened once or more than once?	Pa mwezi wathawu, munganene kuti zinachitika kamozi, kapena pa nthawi zingapo?	1 Once<br>2 More than once	1 Kamodzi<br>2 Koposera kamodzi		
AC49	Physical IPV - YN	ipvphysicyn	In the past 3 months did your partner do the following to you? Slapped, pushed, shoved, hit you with a fist, kicked, dragged, beaten you, choked, burned you, or threatened to use a gun, knife, or other weapon against you?	Pamiyezi 3yadusayi, chbwenzi chanu chapangapo zinthu izi: Kukupasani pama, kukukankhani,kukududani, kukumenyani ndi chibagera, kukumenyani matheche, kuku khwekhwerezani,kukumenyani, kukuopsyezani ndi mfuti kapena mpeni, kapena chipangizo chilichonse?	1 Yes<br>2 No<br>99 Decline to answer	1 Eya<br>2 Ayi<br>99 Akana kuyankha	if no, skip to ipvsexyn	
AC50	Physical IPV - number of times	ipvphysicct	In the past month, would you say this has happened once or more than once?	Pa miyezi 12 imeneyi, munganene kuti zinachitika kamozi, kapena pa nthawi zingapo?	1 Once<br>2 More than once	1 Kamodzi<br>2 Koposera kamodzi		
AC51	Sexual IPV - YN	ipvsexyn	In the past 3 months did your partner do the following to you? Forced you to have sexual intercourse by holding you down or making you afraid of him or forced you to do something sexual that you found humiliating?	Pamiyezi 3 yadusayi, abwenzi anu akukakamizani kuti mugonanenawo, poku kakhirani pansi, kapena kukupangisani mantha , kapena kukukamizani kuti muchite chinthu chokhuzana ndiku gonanana?	1 Yes<br>2 No<br>99 Decline to answer	1 Eya<br>2 Ayi<br>99 Akana kuyankha	if no, skip to ipveconyn	
AC52	Sexual IPV - number of times	ipvsexct	In the past month, would you say this has happened once or more than once?	Pa mwezi wathawu, munganene kuti zinachitika kamozi, kapena pa nthawi zingapo?	1 Once<br>2 More than once	1 Kamodzi<br>2 Koposera kamodzi		
AC53	Economic violence - YN 1	ipveconyn	In the past 3 months did your partner keep you from having the money you needed to buy food or other necessities even when he had money for other things?	Pamiyezi 3 yadusayi, abwenzi anu akukaniza/ kukulepheretsani kukhala ndi ndalama zomwe zimafunikira kuti mukhale ndi chakudya, kapena zofunikira zinazilizonse. Pamene iwo anali nazo ndalamazo.	1 Yes<br>2 No<br>99 Decline to answer	1 Eya<br>2 Ayi<br>99 Akana kuyankha	If no, skip to next section	
AC54	Economic violence - YN 2	ipveconct	In the 12 months prior, were you forced away from your home?	Pamiyezi 12 yadusayi, munayamba mwathamangitsidwapo pa nkhomo panu?	1 Yes<br>2 No<br>99 Decline to answer	1 Eya<br>2 Ayi<br>99 Akana kuyankha		

PS904 - ACASI
STAR Malawi Version 2.0 - 02/05/16

F. End

To be completed by all consenting to participate

Question No.	Construct	Variable name	Wording of question (English)	Wording of question (Chichewa)	Data type	Data type (Chichewa)	Skip	Range
AC55	end time	endtime	Time interview ended		Should enter automatically based on device time			
AC56	Interviewer comments	comments	Interviewer comments on specific questions, respondent, interview		Long text			
